# Mangrove soil as a natural catalyst for green synthesis of silver nanoparticles

**DOI:** 10.3389/fchem.2025.1589836

**Published:** 2025-09-05

**Authors:** Andrea Chacón-Calderón, Juan Miguel Zuñiga-Umaña, Claudia Villarreal, José Roberto Vega-Baudrit, Reinaldo Pereira-Reyes, Yendry Regina Corrales-Ureña

**Affiliations:** 1 National Laboratory of Nanotechnology LANOTEC - National Center of High Technology CeNAT-CONARE, San José, Costa Rica; 2 Academic Master’s in Bioinformatics and Systems Biology, Graduate Program in Biomedical Sciences, School of Medicine, University of Costa Rica, San José, Costa Rica; 3 Escuela de Química, Universidad Nacional de Costa Rica, Heredia, Costa Rica

**Keywords:** Humic acids, nanoparticle synthesis, natural nanoparticles, binding affinity, Pacific, Caribbean

## Abstract

**Introduction:**

Mangrove ecosystems host diverse biogeochemical pathways that enhance their resilience against a wide range of pollutants, from heavy metals to hormones. The unique combination of extreme physicochemical soil conditions and the anaerobic metabolism of mangrove microbiota creates favorable conditions for nanoscale processes.

**Methods:**

The presence of naturally occurring nanoparticles in soil extracts from Costa Rican mangroves at Punta Morales and Cahuita was characterized. Furthermore, we evaluated the ability of these soil extracts to catalyze the formation of silver nanoparticles (AgNPs) under sunlight in saline environments (28°C–31°C within 15 min). Characterization techniques such as transmission electron microscopy (TEM) with selected area electron diffraction (SAED), energy-dispersive X-ray spectroscopy (EDS), Fourier-transform infrared spectroscopy (FTIR), X-ray diffraction (XRD), and UV–Vis spectrophotomery (UV-vis) were used. To investigate the reaction mechanism, we quantified reactive oxygen species (ROS) generated under sunlight and UV light, monitored changes in the absorption bands of the extracts, conducted nanoparticle synthesis in the dark, and measured the reduction potential of the extracts. Ag NPs–extract interactions were evaluated using isothermal titration calorimetry (ITC), and antimicrobial activity was assessed via minimum inhibitory concentration (MIC) assays against *Escherichia coli* (*E. coli*), *Staphylococcus aureus* (*S. aureus*), and *Bacillus subtilis* (*B. subtilis*). Bacterial growth was analyzed using generalized additive models (GAM) and non-parametric tests at specific time points.

**Results:**

Mangrove sediments contained nanoparticles, primarily silicates and carbon-based organic fibers. Soil extracts catalyzed nanoparticle formation, producing mainly Ag NPs and AgCl particles. Pacific extracts showed a higher affinity for the Ag NPs, while Caribbean extracts enabled faster AgNP synthesis due to a higher density of organic binding sites. The proposed mechanism involves organic matter reduction of silver, photolysis, and catalytic ion effects (e.g., iron). Antimicrobial tests revealed species-specific and concentration-dependent responses, with MIC values between 2.5 and 20 μg/mL, depending on bacterial strain and nanoparticle origin. AgNPs synthesized with Caribbean extracts exhibited stronger antimicrobial activity compared to those synthesized with citric acid, highlighting the potential role of humic and fulvic acid coatings.

**Discussion:**

Our findings suggest that mangrove soils naturally harbor nanoscale materials and act as efficient biogenic catalysts for metallic nanoparticle synthesis. The distinct properties of extracts from different mangrove regions influence both the synthesis kinetics and the biological activity of the nanoparticles. This underscores the ecological and biotechnological relevance of mangrove-derived materials.

## 1 Introduction

The natural production of metallic nanoparticles is a widespread phenomenon occurring in various environmental systems (Kaiprath et al., 2024). These nanostructures arise through geochemical cycles and biological activity. Among the least explored ecosystems at the nanoscale level is the mangrove forest, which is a highly productive and biodiverse habitat situated at the interface between the ocean and land. This ecosystem experiences extreme environmental conditions, including high salinity, fluctuating tides, strong winds, elevated temperatures, desiccation, and anaerobic soil environments (Lee et al., 2014; Kaiprath et al., 2024). The diverse microbial, plant, and animal species within mangroves have evolved specialized adaptations to thrive under these conditions, particularly in response to high salinity and low oxygen (Augusthy et al., 2024). With an estimated global biomass of 8.7 gigatons of dry weight, mangrove forests play a crucial role in the carbon cycle, sequestering up to 4.0 gigatons of carbon (Choudhary et al., 2024). Mangrove ecosystems are primarily located between 32° N and 38° S, distributed over 112 countries (Palit et al., 2022). Over the last 2 decades, there has been a 3.4% reduction in mangrove cover worldwide, from 154,000 km^2^ in 1996 to nearly 148,000 km^2^ in 2023 (Bunting et al., 2022). The microbial communities within mangroves facilitate essential nutrient cycling, with the aerobic degradation of organic polymers, such as cellulose, lignin, and pectin, occurring primarily in the uppermost sediment layers (Furusawa, 2019; De Melo et al., 2016; Ding et al., 2025). In deeper layers, anaerobic metabolism prevails, supporting a diverse array of biochemical pathways. Unlike many ecosystems where oxygen-dependent decomposition prevails, sulfate-reducing bacteria are the primary decomposers in mangrove forests, influencing the biogeochemical dynamics of iron, phosphorus, and sulfur (Mo et al., 2023). Nitrogen-fixing, methanogenic, and phosphate-solubilizing bacteria also contribute to nutrient cycling (Das et al., 2024). These microorganisms possess significant biotechnological potential, with applications in biofertilizers, biofuels, bioremediation, and drug discovery (Patra et al., 2020; Booth et al., 2023). However, despite these potential applications, few microbial species within mangroves have been characterized (Palit et al., 2022). Beyond nutrient cycling, mangrove forests also act as natural filters, trapping heavy metals and organic pollutants. Due to their proximity to urban and industrial areas, they receive substantial inputs of contaminants, including polycyclic aromatic hydrocarbons (PAHs), polychlorinated biphenyls (PCBs), and pesticides such as dichlorodiphenyltrichloroethane (DDT), trending to biomagnification (Qiu et al., 2019; Zhang et al., 2019). Heavy metals, including Cu, Zn, Mn, Cd, Cr, Ni, Pb, As, and Hg, readily accumulate in mangrove sediments (Pumijumnong and Uppadit, 2013; Li et al., 2022; Tang et al., 2022). Remarkably, mangrove microbiota effectively manages these pollutants, as anaerobic processes facilitate metal precipitation through sulfide interactions (Das et al., 2022). This prevents bioaccumulation in higher trophic levels, immobilizes metals in sediments, and limits the direct release of contaminants into marine ecosystems. In essence, mangroves serve as physical and biogeochemical barriers against various pollutants introduced by human activity. With the increasing application of nanotechnology in industries and agriculture, the generation of nanometric waste has emerged as a new environmental concern (Vaish and Pathak, 2023). A significant component of mangrove soils, humic substances, constitutes approximately 80% of the organic matter (Mathew et al., 2021). These complex organic macromolecules, derived from the degradation of plants and microorganisms, are classified into humic acids, fulvic acids, and humin based on their solubility properties (Gupta et al., 2021). Their unique chemical characteristics have attracted attention in diverse fields, including medicine, cosmetics, and environmental remediation. Humic acids, for example, maintain a negative charge in neutral and alkaline pH environments, allowing them to bind to cationic sites on virus surfaces and inhibit replication. Their role as mutagenesis inhibitors, both inside and outside cells, has been documented (Taherkhani and Piri, 2023). Their capacity to absorb UV-visible radiation has led to applications in skincare and sun protection products, with additional benefits from their antioxidant properties (Da Silva et al., 2018; Parisi et al., 2023). Moreover, humic acids can act as solubilizing agents, facilitating the delivery of pharmaceutical and cosmetic ingredients that are naturally insoluble in water. Humic substances are highly effective adsorbents in environmental applications, capturing both organic and inorganic pollutants. Their flexible structures and numerous adsorption sites allow them to “trap” contaminants. At the same time, their reducing properties enable the transformation of metal species from toxic to less harmful oxidation states, even promoting nanoparticle formation (De Melo et al., 2016; Kučáková & Pavlíková et al., 2017). The interaction of metal ions with natural organic matter (NOM) and reactive oxygen species plays a fundamental role in the environmental formation of metallic nanoparticles. The predominant functional groups in humic acids—carboxylic and phenolic moieties—enhance metal complexation, influencing ion mobility and bioavailability. While the remarkable capacity of mangrove forests to retain heavy metals is well established, their potential role in nanosynthesis remains largely unexplored. Various plants and microorganisms commonly found in mangrove forests have been utilized to develop nanomaterials, often with a focus on obtaining nanoparticles (Kathiresan et al., 2012; Narendran and Kathiresan, 2016; Vaish and Pathak, 2023). The chemical mechanisms leading to nanoparticle formation vary widely, involving spontaneous reactions or processes triggered by thermal and photochemical energy (Sharma et al., 2019; Sharma et al., 2015; Glover et al., 2011). This study aims to assess the potential of Costa Rican mangrove ecosystems for nanoparticle synthesis, using silver nanoparticles (AgNPs) as a model system. Silver is a good model nanoparticle because its characterization is well known, visually responsive, biologically active, and relevant to many real-world applications. It serves as a benchmark in nanomaterials research, helping scientists optimize processes that can later be adapted for other, more complex or specialized nanomaterials. AgNPs are widely used in consumer products, including textiles, cosmetics, coatings, water filters, and wound dressings, due to their antimicrobial properties.

By comparing the elemental composition, salinity, and organic matter content of sediments from different mangrove regions and depths, we evaluate their ability to catalyze nanoparticle formation. Additionally, we examine the types of nanostructures in sediments using characterization techniques such as transmission electron microscopy (TEM), energy dispersive X-ray spectroscopy (EDS), X-Ray diffraction (XRD), ultraviolet – visible spectrophotometry (UV-Vis), cyclic voltammetry (CV) and Attenuated Total Reflection Fourier Transform Infrared (ATR-FTIR) to assess the catalytic capacity of extracted natural organic matter for nanoparticle formation. The antimicrobial properties of AgNPs synthesized using mangrove extracts are further analyzed through minimum inhibitory concentration (MIC) tests against *E. coli*, *S. Aureus*, and *B. subtilis* to determine the impact of organic matter coatings on nanoparticle bioactivity.

## 2 Experimental procedure

### 2.1 Mangrove sediment filtrate

**FIGURE 1:**
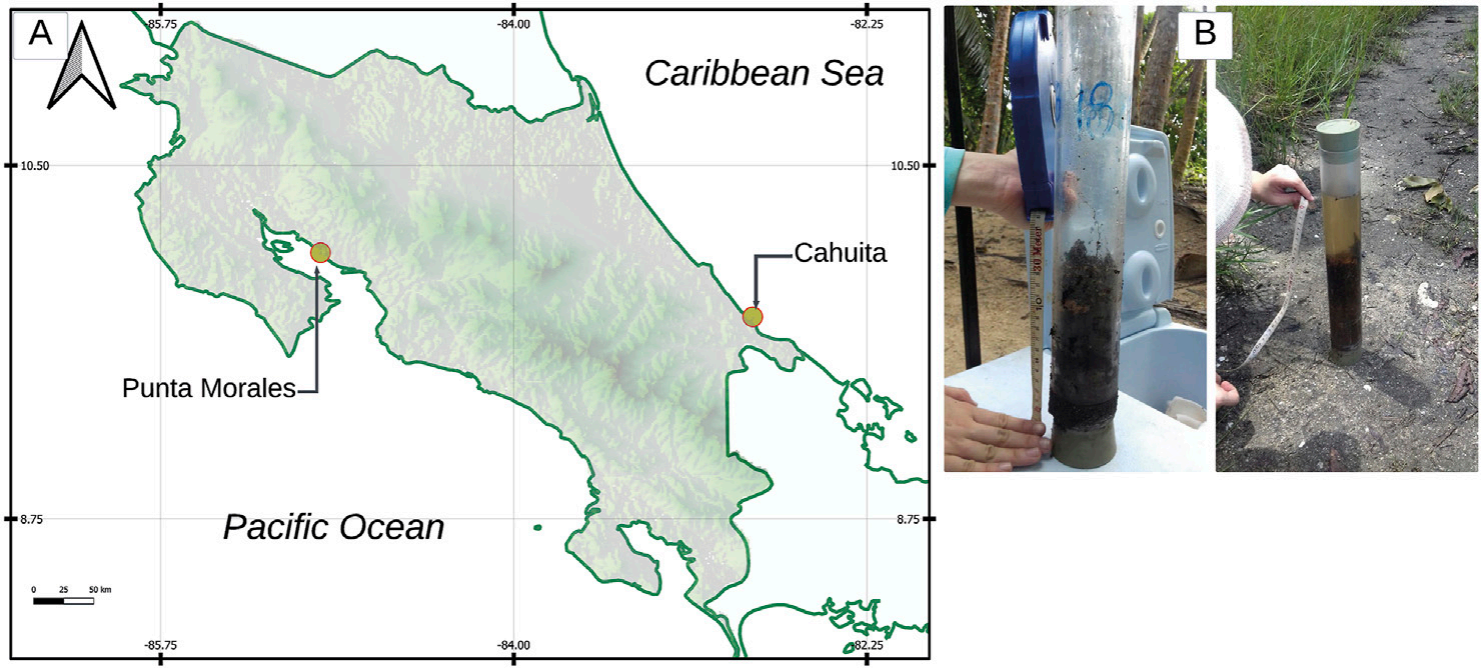
**(A)** The sample collection sites are Punta Morales (top left) and Cahuita (top right). **(B)** Photographs show the mangrove soil sampling process using acrylic tubes.

The collection of mangrove soils was conducted at two geographical points in Costa Rica. The first, on the Atlantic coast, corresponds to two mangrove associations in Cahuita National Park: at 400 m from the beginning of the trail to Punta Cahuita. The second was on the Pacific coast, in the mangrove areas along the shore of the Estero Morales, adjacent to the National Marine Coastal Science Station facilities. Samples were taken using acrylic tubes, fitted with a steel tip, provided by the Center for Research in Marine Sciences and Limnology (CIMAR, Costa Rica). The standard procedure for sampling this type was followed, which consisted of forcefully inserting the tubes into the soil as far as the terrain allowed (given the presence of roots that block the tube), securing rubber stoppers in the upper opening to create a vacuum, and then extracting the tube, thus obtaining a soil column in each tube. Photographs of some of the obtained samples are shown in Figure 1. Once collected, the samples were stored in a cooler for transport to the laboratory, where they were refrigerated at 4°C. Once in the laboratory, the soil samples were separated by depth level. Soils from 0 to 10 cm deep were categorized as type surface soils (S), and those from 10 to 20 cm as deep soil are considered inner soil (I). A composite sample was created for each soil type, mixing different samples from the same depth and sampling location (a minimum of five individual samples comprise one composite sample). Thus, the process concluded with four composite samples. The samples were filtered to remove excess water from the muddier soils and then allowed to dry, first in the air and then in an oven at 40°C. Once dry, they were passed through a 2 cm sieve to separate leaves, roots, and larger stones.

### 2.2 Soil analysis

100 g of each sample were sent to the Laboratory of Soils, Foliage, and Waters at the National Institute for Agricultural Technology Innovation and Transfer (INTA, Costa Rica), where the following analyses were performed: complete chemical analysis to determine the concentration of Ca, Mg, K, P, Fe, Zn, Cu, Mn, S, and N; determination of pH, acidity, and electrical conductivity, determination of organic matter and determination of texture and particle size. The extraction method used for Ca, Mg, Fe, Cu, Zn, and Mn was the Olsen KCl method (1:10, soil: KCl solution). The concentration was determined using atomic absorption spectroscopy using a Perkin-Elmer Analyst 700. K was extracted using the same procedure, and its concentration was determined using a UV-Vis spectrometer (Jasco V-700). Soil pH is measured using a pH meter in a soil-water suspension, following protocols outlined in standard soil analysis references. Electrical conductivity (EC) was measured to assess soil salinity levels, using a conductivity meter in a soil-water extract. A dry combustion method was employed to determine the organic matter, and a CHN analyzer was utilized. The texture and particle size were determined using the hydrometer method. This technique measures the relative proportions of sand, silt, and clay based on sedimentation rates in a soil suspension.

### 2.3 Preparation of mangrove soils extracts

Each soil sample was mixed with distilled water to obtain a soil concentration of 25 mg/mL, following the concentrations suggested by Yin et al. (2012). The solutions were agitated for 24 h and then centrifuged at 2,200 g for 15 min. The supernatant was passed through a 0.2 µm (Minisart NML celulose acetate, Sartorius, Germany) filter and stored refrigerated at 4°C. For the ATR-FTIR analysis of the extracts and the generated nanoparticles, the samples were previously lyophilized. A LABCONCO freeze dryer, model 7753020, was used for this process, in which the samples were cooled to a temperature of −40°C and a pressure of 13.3 Pa.

### 2.4 Synthesis of natural silver nanoparticles

The synthesis of silver nanoparticles under solar irradiation was performed by modifying the procedure described by Yin et al. (2012). Silver nitrate with a purity of greater than 99.0% was purchased from Sigma Aldrich. The reagents were used without further purification. Lower concentration solutions were prepared from the stock extracts of each soil sample: for the Cahuita extracts, the final concentration was 5 g L^−1^, while for the Punta Morales samples, it was 0.5 g L^−1^. Subsequently, silver nitrate was added to the filtrate to achieve a final concentration of 0.1 M AgNO_3_. The pH levels of the extract solution and the silver nitrate was 5.53, 6.73, 6.53, and 6.69 for Punta Morales Inner (PMS), Punta Morales surface (PMS), Cahuita inner (CI) and Cahuita surface (CS), respectively. The samples were exposed to sunlight in glass vials for 15 min. The incident light intensity was measured using a digital Lux meter (LX 1330B, China), as shown in Supplementary Figure S1. The reaction temperature varied between 28°C and 31°C during the experiments. Each experiment was repeated at least 3 times. After the determined exposure time, the vials were covered with aluminum foil, and then the solutions were immediately centrifuged at 13,000 rpm in a microcentrifuge (Labnet Spectrafuge 16M). The pellet was rinsed three times with MilliQ water and refrigerated at 4°C. As control groups, the same experiments were conducted with the following variations: a) same preparation but without sunlight exposure, b) extract only, and c) AgNO_3_ only exposed to sunlight for up to fifteen minutes. Fifteen minutes was chosen as the endpoint to avoid the precipitation of larger particles and aggregation due to particle growth. This allowed us to follow the reaction kinetics and perform the antimicrobial analysis with similar well-dispersed NPs. To calculate the amount of AgNPs per sample, as it was mixed with organic material not bound to the NPs, the NPs were dialyzed using a membrane with a 10 kDa cut-off (Membra-Cel Viskase, United States) in MilliQ water for 24 h. The remaining solution, which we expected to be depleted of Ag^+^ ions and not form insoluble particles, was lyophilized. EDS analysis was performed to determine the amount of Ag and Cl in the sample. Based on this analysis, Ag that was expected to be part of AgCl, which is insoluble, was subtracted from the total Ag amount to calculate the concentration of Ag NPs. Additionally, the TGA of the lyophilized materials was performed to subtract the amount of organic material (degradation up to 500°C, Supplementary Figure S2) from the inorganic fraction.

### 2.5 Mechanism of nanoparticle formation

To gain deeper insight into the possible mechanisms involved in nanoparticle formation, five experiments were performed:

(I) To assess the redox behavior of the organic matter and determine its potential to act as a reducing agent in nanoparticle formation, CV measurements were performed. Electrochemical experiments were conducted using a CORRTEST electrochemical workstation (Wuhan Corrtest Instruments Corp., China), equipped with a conventional three-electrode system. The working electrode was made of glassy carbon, the counter electrode was a platinum wire, and the reference electrode was an Ag/AgCl electrode (saturated KCl). The CV measurements of the extracts were performed in the range between −0.55 and 0.85 V, starting with a cathodic potential, at a scanning rate of 100 mV/s. Three consecutive scans were recorded for each sample. To ensure reproducibility and a clean electrode surface prior to each experiment, the platinum electrodes were polished with 0.05 μm alumina slurry and rinsed thoroughly with deionized water, then sonicated with acetone, rinsed with isopropyl alcohol and dried with pressurized air. The electrodes were then electrochemically etched at 2 V vs Ag/AgCl for 600s, followed by 50 voltammetric cycles between −0.191 a 1.139 V vs Ag/AgCl. The oxidation potential of electroactive species in the organic extract was identified from the anodic peaks in the cyclic voltammograms. These data were used to evaluate the presence of reducing agents capable of driving the formation of silver NPs.
(II) To evaluate whether photoinduced reactions could be involved, the absorbance correlated with nanoparticle formation at 300 nm was measured, comparing the synthesis in the dark (at 23°C–25°C), standard white laboratory light, and sun irradiation after 15 min.
(III) To investigate whether complexes formed between ionic silver and organic matter could promote charge transfer and drive the direct reduction of silver. This was assessed by analyzing shifts in the UV-Vis absorption spectrum of the organic matter, with a peak at 300 nm, upon mixing 3 mL of the extract (2 mg/mL) with 10 μL of AgNO_3_ (10 mg/mL) and reading quickly after mixing in the UV cell under ambient conditions.
(IV) To test whether photolysis of organic matter could generate reactive oxygen species (ROS), such as superoxide radicals (O_2_•^−^), via reactions involving phenolic groups and dissolved oxygen, the extracts were irradiated with UV light for 20 min under the same conditions used in the synthesis. ROS generation was quantified using Acetic 5-(chloromethyl)-2-(3,6-diacetoxy-2,7-dichloro-9H-xanthen-9-yl)benzoic anhydride (CM-H_2_DCFDA, Invitrogen, Thermo Scientific, United States), a fluorescein-based probe commonly used as an indicator of ROS. Immediately after irradiation, 100 μL of the extract was placed into wells of a 96-well microplate and mixed with 10 μL of the probe at a final concentration of 500 μM. The samples were incubated in the dark at 37°C for 30–60 min to allow the probe to react with any ROS present. Non-irradiated extract and probe-only in water were used as negative controls, while 0.02% (w/v) H_2_O_2_ (Sigma Aldrich 30 wt% % reagent, Germany) served as a positive control. The samples were irradiated using a Fluorescence Analysis Cabinet Model CM-10A (Spectroline, United States). Fluorescence was measured in triplicate using a microplate reader (BioTek Synergy H1 microplate reader controlled by Gen5 software, Winooski, VT, United States) with excitation/emission settings of 495/525 nm. An increase in fluorescence intensity compared to the non-irradiated control was used as an indicator of ROS generation promoted by UV exposure in the natural extract.
(V) The synthesis of the nanoparticles involved the addition of Fe(NO_3_)_3_ (98%, Sigma Aldrich, United States) at a final concentration of 0.025 M to the reaction for 20 min, to evaluate whether other metals, such as iron, could enhance nanoparticle formation. To assess this, nanoparticle concentration was determined using a calibration curve prepared with NIST 8017 (US Department of Commerce, United States) (75 nm) reference nanoparticles; the curve obtained was y = 0.61611X + 0.50024 (R^2^ = 0.97). To each solution, the control absorbance at time 0 was subtracted to calculate the concentration.

### 2.6 Characterization

#### 2.6.1 Attenuated total reflectance fourier transform infrared spectroscopy (ATR-FTIR)

A Thermo Fisher Scientific Nicolet 6,700 model was used for infrared spectroscopy analysis, with a scan range from 500 to 4,000 cm^−1^. The obtained spectra were analyzed using ThermoFisher’s OMNIC and OriginLab’s Origin software.

#### 2.6.2 Ultraviolet-visible spectrophotometry (UV-Vis)

UV-vis analysis was performed using a Shimadzu UV-1800 model, with scans from 800 nm to 190 nm at a resolution of 1 nm, in 1 cm quartz cuvettes. The obtained spectra were analyzed using Shimadzu’s UV-Prove software and OriginLab’s Origin software.

#### 2.6.3 Isothermal titration calorimetry (ITC)

Isothermal titration calorimetry was analyzed using a Nano ITC (TA Instruments, USA). The cell was filled with the standard silver nanoparticles synthesized using citrate solution (AgNPs@citrate), the mangrove soil extract, or its corresponding blank. Each titration consisted of 20 injections of 5 µL each, administered at 300-s intervals, with a stirring speed of 350 rpm and a temperature of 25°C. The concentrations in the syringe and cell are described in Table 1. Before starting the titration, the calorimeter was equilibrated for the necessary time to achieve a baseline deviation of less than 1 μJ/s. Baseline data were collected for 300 s before the first injection and after the last injection. The compounds in the syringe or the cell were optimized to ensure that the heat flow remained within the instrument’s detection range and that a single injection did not saturate the system. For the modelling, the first titration injection was not taken into account.

**TABLE 1:** Concentration of the mangrove extract and silver nanoparticles used in the ITC experiments.

Concentration in syringe (mg/mL)	Concentration in cell (mM)
6.85 CI	0.1752 (AgNPs@citrate)
64.21 CS	0.1853 (AgNPs@citrate)
7.11 PMI	0.4633 (AgNPs@citrate)
5.86 PMS	0.4633 (AgNPs@citrate)
10 mM (AgNPs@citrate)	6.34 Humic acid control
10 mM (AgNPs@citrate)	9.64 citric acid

#### 2.6.4 Dynamic light scattering (DLS)

A dynamic light scattering instrument (N SZ-100V2, Horiba) measured the size distribution and zeta potential. Nanoparticle solutions containing a concentration of 0.1 mg/mL were analyzed. Also, the control mangrove extracts before adding the AgNO_3_.

#### 2.6.5 X-ray diffraction (XRD)

The XRD patterns were recorded in an Empyrean diffractometer using Cu K radiation in the 5°–90° range in steps of 1° in 24 s. The XRD spectra presented were obtained for the sample of Cahuita 0–10 cm, which produced more nanoparticles in a shorter time.

#### 2.6.6 Transmission electron microscopy (TEM)

TEM micrographs and selected area diffraction (SAED) diffraction images were obtained on a JEOL JEM-2010 TEM coupled with an EDS detector set at 120 kV, using magnifications between 250,000X and 300,000X. Ganta 794 Digital Micrograph software was used for data adquisition. The sample was mounted onto a copper/palladium 400 mesh formvar-coated grid and allowed to dry. In the case of the mangrove samples, the solutions were sonicated for 10 min in an ultrasonic bath to dissolve any unstable particle. Samples from the solutions before and after ultrasonication were analyzed to determine changes due to this process.

### 2.7 Antimicrobial properties

For the MIC assay, the procedure described by Kadeřábková et al. (2024) was followed. The entire process was conducted under aseptic conditions in a biosafety cabinet. Three bacterial species were cultured: *Bacillus subtilis* ATCC1174, *Staphylococcus aureus* ATCC25923 (both Gram-positive), and *E. coli* ATCC25922 (Gram-negative). An inoculum was taken using a loop and cultured in Mueller-Hinton broth for approximately 16 h. The OD_600_ was measured (ThermoScientific UV-Vis Spectrophotometer) in a sterile 0.85% m/v NaCl solution for each bacterial species prior to inoculation, and the absorbance was adjusted to 0.002 (∼1 × 10^5^ CFU) to inoculate 50 µL into each well, with a final volume of 100 µL.

Serial dilutions at five concentration levels, starting from 0.02 mg/mL, were prepared in microplates for both extracts and nanoparticles, according to the following treatments: CI, CS, PMI, and PMS. The microplates were incubated for 20–24 h in a BioTek Epoch 2 Microplate Spectrophotometer (Agilent Technologies, United States) at 37°C with readings taken every hour at 600 nm. Orbital shaking was applied for 30 s prior to each reading. Controls included a positive control (1× MH medium + bacteria), 0.1 mM AgNO_3_, and AgNPs at a maximum concentration of 0.02 mg/mL. Concentration (RM 8017, AgNPs NIST standard ∼75 nm), and a negative control (1× MH medium without bacteria).

Optical density (OD_600_) readings from the microplate assays were normalized using the pre process and predict functions from the caret package in R (Kuhn, 2008). To identify and validate differences in bacterial growth curves over time across the various synthesis treatments—namely nanoparticles synthesized from mangrove samples from Punta Morales or Cahuita, and from different soil strata (Surface/inner)—generalized additive mixed models (GAMs) were constructed using the mgcv package (v1.9-1) in R (Wood, 2011). These models employed an identity link function with a Gaussian error distribution.

Given the high number of hypothesis tests required by the experimental design and data structure, p-values for group contrasts were adjusted using the Holm method to control for Type I error. Bacterial growth trends were visualized using the ggplot2 package (v3.5.1), employing local regression smoothing (LOESS) to capture dispersion patterns (Wickham, 2016).

To assess the statistical significance of pairwise contrasts, the glht and confint functions from the multcomp package (v1.4-28) were used. For the analysis of the joint effects of treatment type (extracts or nanoparticles from distinct mangrove sources) and concentration on bacterial growth, OD_600_ values between 15 and 20 h were extracted. Due to non-normality in the data (W = 0.8, p < 0.01), the nonparametric Scheirer-Ray-Hare test was applied using the rcompanion package in R (Mangiafico, 2025). Post hoc comparisons were conducted using Dunn’s test, with p-values corrected via the Benjamini-Hochberg method (R, FSA package; Ogle et al., 2025).

## 3 Discussion

Table 2 shows the elemental composition of the mangrove sediments. It is noted that the soils of Punta Morales (Pacific) are richer in potassium, magnesium, iron, and manganese than those of Cahuita (Caribbean). At the same time, they show similar levels of phosphorus, copper, zinc, and sulfur. Calcium is the only ion with a significantly higher concentration in Cahuita than in Punta Morales, as well as the total carbon content.

**TABLE 2:** Elemental composition of the mangrove soils measured by atomic absorption.

Sample	K	Ca	Mg	P	Fe	Cu	Zn	Mn	S	% N	% C total
	(cmol L^−1^)			(mg kg^−1^)							
Cahuita 0–10 cm (CS)	0.39	9.9	2.8	10	38	2	5.0	25	11.0	0.18	8.50
Cahuita 10–20 cm (CI)	0.48	9.4	4.0	8	36	3	4.3	28	9.0	0.15	8.03
Punta Morales 0–10 cm (PMS)	1.35	6.7	9.7	9	177	1	7.5	87	17.0	0.04	3.72
Punta Morales 10–20 cm (PI)	1.16	5.2	9.9	9	124	1	9.0	103	15.0	0.02	2.48

Table 3 presents the pH values of the sediments, showing that soil pH is significantly lower in Punta Morales compared to Cahuita. The high total carbon content and pH above 7 in the Cahuita soils suggest a higher calcite content (Sulpis et al., 2022). Studies have shown an interdependence between pH and calcium concentration, indicating that under high pH and elevated calcite levels, the biodegradation of molecules such as pesticides is enhanced (Warton and Matthiessen, 2005). The electrical conductivity of the soils—a parameter correlated with salinity—is higher in Punta Morales than in Cahuita, as is the exchangeable acidity. Mangrove soils tend to be highly saline not only because they are located in transitional ecosystems (at the interface of freshwater and saltwater), but also due to the salt-exclusion mechanisms in mangrove roots, which increase local soil salinity (Krishnamurthy et al., 2014).

**TABLE 3:** Characterization of mangrove soils: pH, electric conductivity, acidity, % of organic matter and C/N.

Sample	pH	Electric conductivity (ms cm^−1^)	Acidity (cmol L^−1^)	% Organic matter	C/N
Cahuita 0–10 cm (CS)	7.0	1.6	0.1	8.0	46.3
Cahuita 10–20 cm (CI)	7.3	2.6	0.1	7.5	54.2
Punta Morales 0–10 cm (PMS)	4.2	16.4	0.7	8.3	105.2
Punta Morales 10–20 cm PMI	3.2	13.6	4.1	5.7	124.1

**FIGURE 2:**
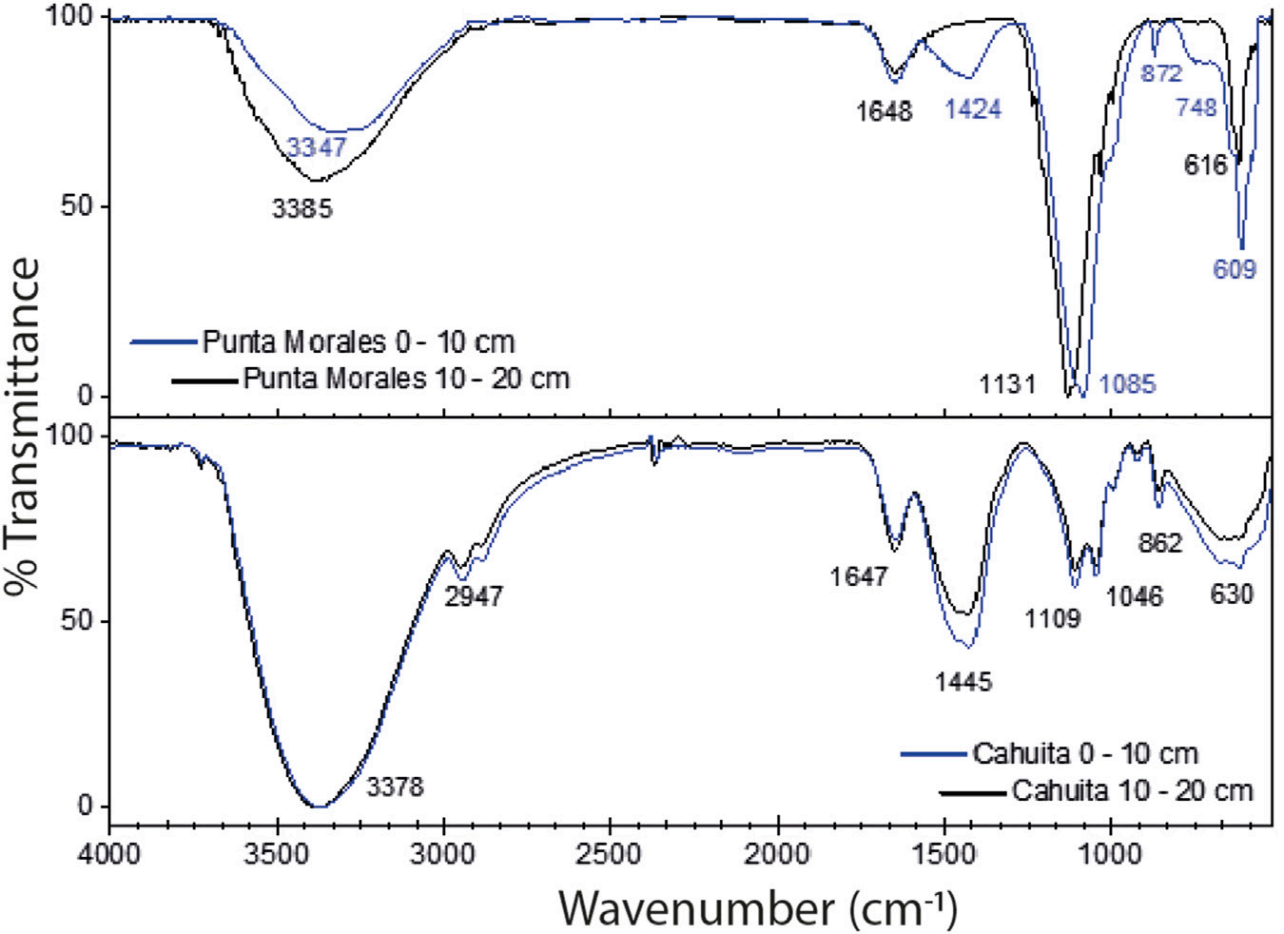
ATR-FTIR spectra of lyophilized aqueous extracts from Punta Morales and Cahuita, collected at soil depths of 0-10 cm (surface) and 10-20 cm (inner).

ATR-FTIR enables the identification of primary and secondary minerals, including silicates, clays, and aluminosilicates. It also enables the determination of organic matter, biomass, and humic substances by identifying functional groups in carbohydrates, lignins, cellulose, lipids, and proteins. Figure 2 shows the spectra obtained from the ATR-FTIR analysis of the lyophilized extracts. Similar peaks are present in all spectra, regardless of the site of origin. The first is the broad band centered between 3,400 and 3,300 cm^−1^, commonly associated with stretching O-H bonds in alcohols and N-H bonds in amines. The peaks around 1,100 cm^−1^ are related to the stretching of C-O bonds in carbohydrates and alcohols; they are also characteristic of the Si-O stretching of aluminosilicates present, especially when combined with the bands between 860 and 870 cm^−1^. In the spectra from Cahuita, the bands at 2,947 cm^−1^ and 1,445 cm^−1^ indicate the presence of aliphatic chains; the second band is also present at 1,424 cm^−1^ for the surface soil extract from Punta Morales, although the shift to lower wavenumbers could mean it is overlapped with the deformation of O-H bonds and the stretching of C-O bonds in phenolic groups. The difference in the intensities of certain bands indicates the difference in organic matter between the soils. For example, the presence of bands around 2,900 cm^−1^ for the extracts from Cahuita, as well as the higher intensity of the bands at 1,647 cm^−1^ and 1,445 cm^−1^, are properties that are more prominent in humic acids, which have more polyaromatic and substituted structures than fulvic acids (Tatzber et al., 2008). Additionally, the presence of the band at 1,424 cm^−1^ (associated with humic acids) for the more surface soil extract from Punta Morales but not for the deeper one aligns with the consideration that, due to the pH of the extract, the former would consist of a mixture of humic and fulvic acids, while the deeper one would be mainly composed of fulvic acids. It is essential to mention that none of the spectra show the most typical bands for the COO- group, either in its deprotonated form or as a carboxylic acid (2,600 cm^−1^, 1720 cm^−1^, or 1,385 cm^−1^). These groups are essential in soil organic matter, as they are among the main ones interacting with the environment’s metals and other reactive species. The absence of these peaks in the spectra suggests that the extraction method yields very low concentrations of these species. This point is even more precise when comparing the obtained spectra with the infrared spectrum of a standard humic acid (Supplementary Figure S3). The characteristics of the extracted material are presented in Table 4. The amount of Cl- ions was measured to prepare the control solutions. The salinity of the extract is also an essential factor; Kida et al. (2017) determined that as the electrical conductivity of the extracting solution increases, humic acids tend to precipitate.

**TABLE 4:** pH and Cl^−^ concentrations in mangrove soils extracts (25 g·L^−1^), filtered through 0.2 μm filters, and hydrodynamic radius of natural nanoparticles in the mangrove extracts measured by DLS.

Sample	pH (21°C)	Concentration Cl^−^(mM)	Hydrodynamic radius (nm)
Cahuita 0–10 cm (CS)	7.66	0.07	75.8 ± 33.9^a^
Cahuita 10–20 cm (CI)	7.70	1.03	81.7 ± 114.2^a^
Punta Morales 0–10 cm (PMS)	6.17	11.2	164.6 ± 3.60^b^
Punta Morales 10–20 cm (PMS)	5.58	8.7	257.68 ± 68.65^b^

^a^ 0.06% Tween 20 was added to improve dispersion.

^b^ AgNO_3_ + mangrove extract reaction time 0.

**FIGURE 3:**
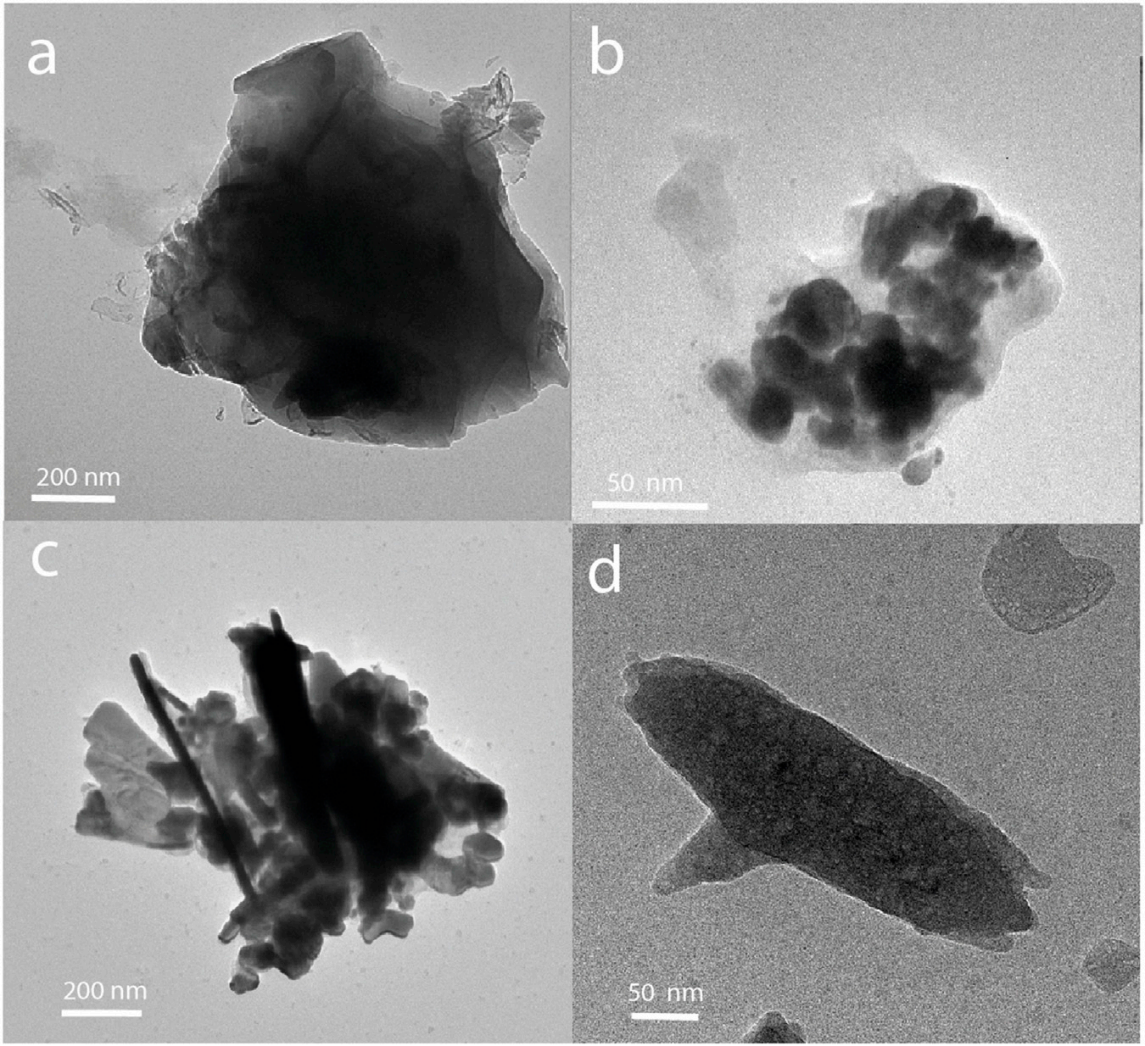
TEM micrographs of natural nanoparticles found in mangrove extracts: **(a)** 0–10 cm depth) and **(b)** 10–20 cm depth from Punta Morales; **(c)** 0–10 cm depth and **(d)** 10–20 cm depth from Cahuita.

Characterizing mangrove extracts using electron microscopy enables the correlation of compositional analysis with the morphology of the species. Table 5 presents the atomic composition of six different particles (three from the uppermost soil layer and three from the deepest layer) collected from the Cahuita and Punta Morales mangroves. Figure 3 shows examples of TEM micrographs of various nanostructures found in the extracts. Pre-existing nanoparticles in the mangroves, considered natural nanoparticles, display different morphologies (Figures 3a–d). In some cases, patterns from dried salts may also be present; however, the common fractal patterns are generally not observed as a major feature of these samples. Figure 3b shows electron-dense particles—typically associated with metallic species—embedded in amorphous material that was not removed during dialysis. DLS analysis of the native extracts from Cahuita and Punta Morales, performed prior to synthesis (time = 0), confirmed the presence of nanoparticles with hydrodynamic radius consistent with those observed in the TEM images. These findings indicate that the nanoparticles were present in solution, not artifacts resulting from the drying process, and that they had not dissolved before the reaction began (see Table 4).

**TABLE 5:** Elemental atomic composition (at%) of natural nanoparticles found in the soils extracts of the Cahuita and Punta Morales, characterized by TEM-EDS.

Sample		Elemental composition (at %)									
		C	O	Na	Mg	Si	Al	S	Cu	Fe	Ca
CS	NNP 1	78.6	20.7	-	0.1	0.5	0.1	-	-	-	-
	NNP 2	-	53.2	27.9	2.7	10.4	1.8	-	-	-	-
	NNP 3	54.9	41.7	0.4	0.3	2.1	0.3	-	-	-	-
CI	NNP 4	65.4	32.4	-	-	1.8	-	-		-	-
	NNP 5	51.1	47.3	-		0.8	0.6	-	0.7	0.2	-
	NNP 6	46.8	51.0	-	0.1	0.5	0.1	-	0.1	0.2	-

Sample		Elemental composition (at %)										
		C	O	Na	Mg	Al	Si	P	S	Ca	Fe	Cu
PMS	NNP 1	37.1	55.6	1.2	0.3	0.8	4.7	0.2	-	0.1	-	0.2
	NNP 2	45.9	47.2	2.9	0.8	0.1	2.7	0.1	0.1	0.1	0.1	0.1
	NNP 3	49.0	44.0	3.3	0.7	0.1	2.5	0.1	0.1	0.1	0.1	0.1
PMI	NNP 4	57.9	25.0	6.7	-	-	0.5	-	1.9	0.1	-	1.1
	NNP 5	67.7	28.1	3.7	0.1	-	0.3	-	0.1	0.1	-	0.1
	NNP 6	78.6	20.7	-	0.1	0.1	0.5	0.1	-	0.1	-	0.1

The mangrove natural nanoparticles are highly polydisperse, forming agglomerations and emulsions with the soil’s organic material. The sodium content is higher for natural Cahuita particles, which, together with the silicon content, suggests that these particles consist of sodium and magnesium silicates. It is essential to note that the structures of Cahuita contain a significantly higher amount of sulfur than those of Punta Morales, as well as higher calcium levels. The high presence of calcium is to be expected considering that the calcite found at a macroscopic level in the soils of Cahuita can form CaCO_3_ nanoparticles because of erosion and weathering (Smita et al., 2012), or the metal containing NP could be presented in the filtrate as part of the natural nanoparticles produced in the ecosystem by microorganisms (Kathiresan et al., 2009). By comparing the structures at the two soil depths for Punta Morales, between 10 and 20 cm deep contain a smaller amount of carbon but a higher concentration of oxygen and several metals, including aluminum, magnesium, and iron. Materials such as quartz, feldspars, gibbsite, kaolinite, illite, and vermiculite, have been previously reported, but not the nanoparticles (Souza, 2008).

### 3.1 Synthesis of nanoparticles using mangrove soil extracts

**FIGURE 4:**
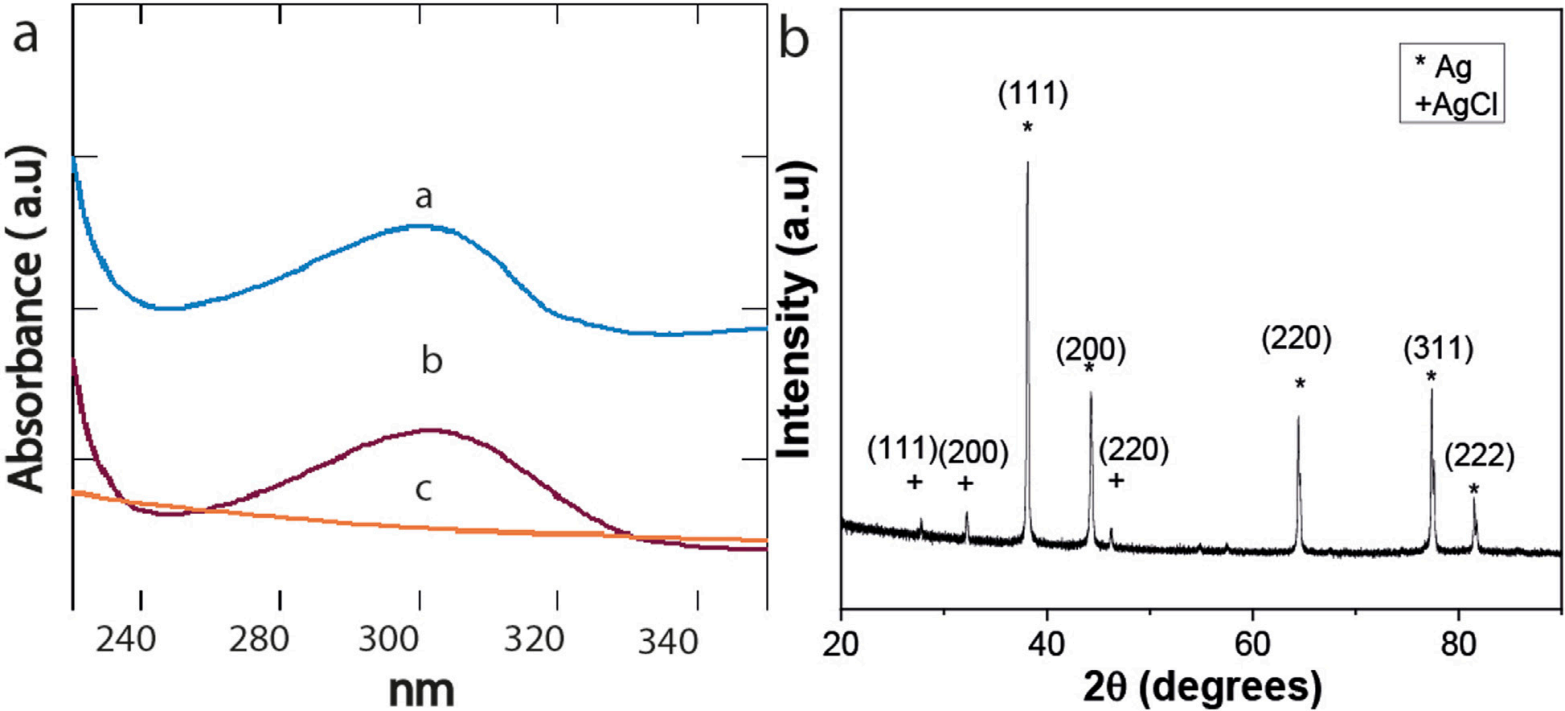
UV spectra of **(a)** Mangrove-AgNPs synthesized by light, **(b)** AgNO_3_ exposed to light, **(c)** PMS mangrove extract. XRD spectra of Ag NPs synthesized by mangrove sediments and sunlight from PMS.

Changes in the UV absorption spectra were monitored to determine the formation of colloidal silver, as indicated by visible absorption at 250–350 nm in the salt-mangrove filtrate solutions, Figure 4a. The XDR difractogram of the particles formed in a higher amount, synthesized with the PMS extracts, are shown in Figure 4b. The characteristic surface Plasmon resonance SPR was observed by UV-vis between 300 nm and 310 nm. There is no evidence of an absorption peak at the 300–310 nm region for the mangrove filtrate, as we will discuss below, and lower intensity for the AgNO_3_ in water exposed to light in the same conditions as ; as the salt concentration decreased due to the precipitation of AgCl. The color of the solution was also indicative of NPs formation (Supplementary Figures S4–S6). XRD analysis of the AgNPs material showed peaks at 38.1°, 44.3°, and 64.5°, 77.4° and 81.7° which can be indexed with the planes (111), (200), (220), (311) and (222) for the face-centered cubic (fcc) structure of metallic silver (Wang et al., 2020), Figure 4b. The peaks corresponding to AgCl are present, indicating that the nanoparticles obtained are primarily composed of Ag^0^ and AgCl (Figure 4b).

**FIGURE 5:**
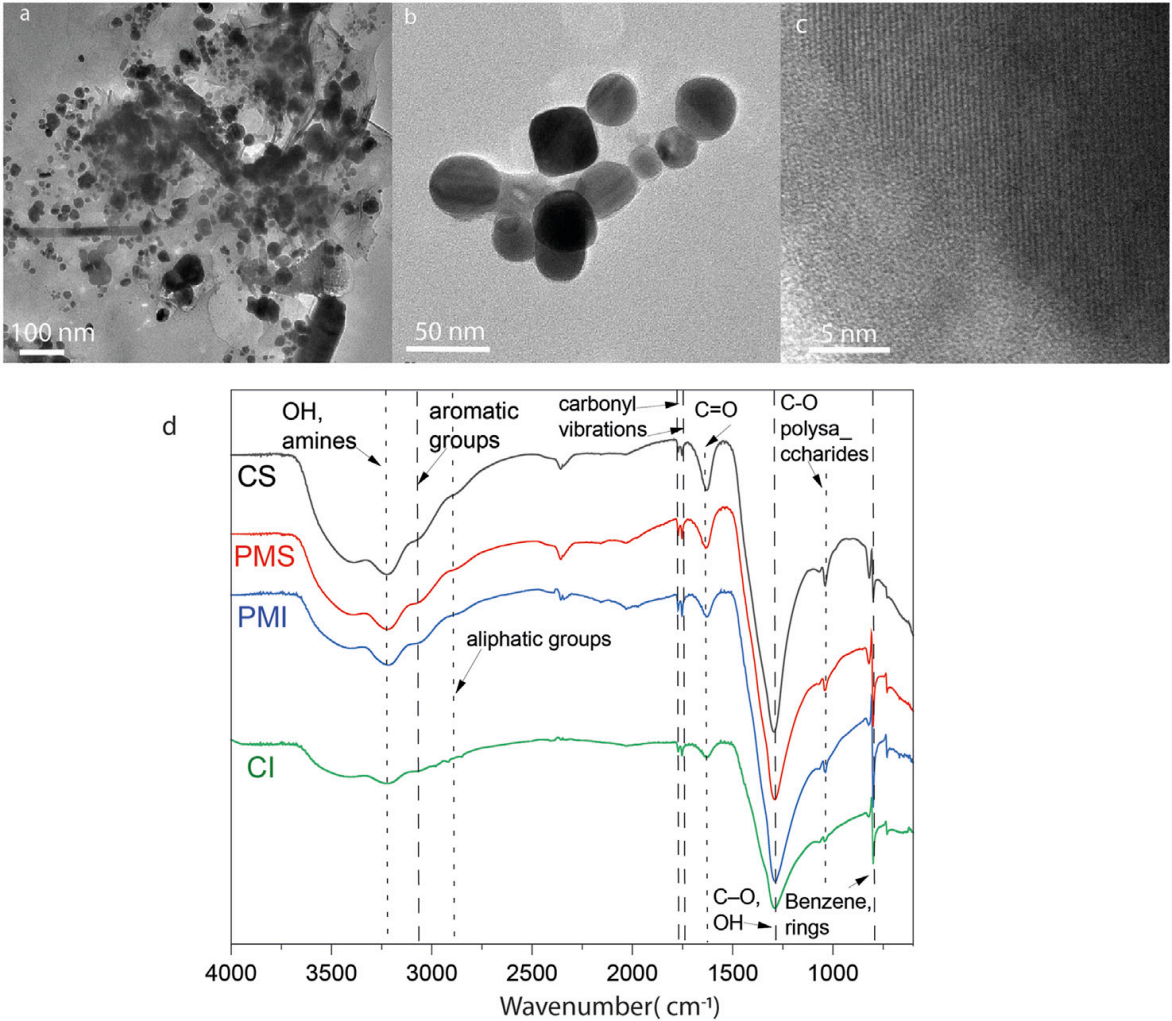
**(a–c)** TEM micrographs of Ag nanoparticles obtained from the mangrove filtrate from Punta Morales. **(d)** ATR-FTIR of the NPs.

**FIGURE 6:**
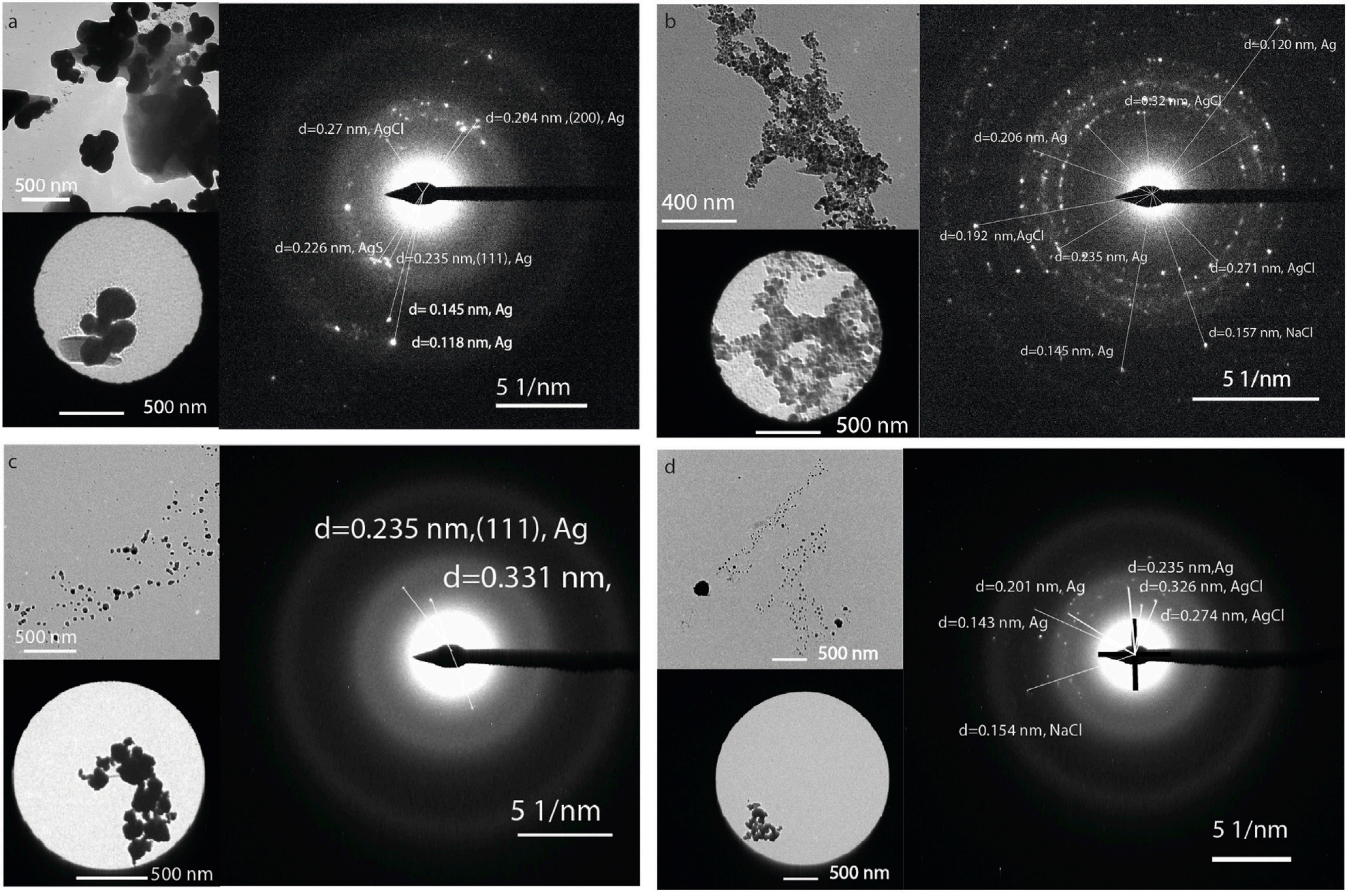
TEM and selected area diffraction image of the NPs synthesized using the extract from **(a)** CI, **(b)** CS, **(c)** PMI, and **(d)** PMS.

Figures 5a,b show roundish and hexagonal morphologies; the nanoparticles tended to aggregate and bind together through organic material, Figure 5c. The EDS analysis revealed a heterogeneous composition that can be attributed to the presence of different metals in the nanoparticles isolated from the mangrove filtrate. However, the XRD analysis indicates that the main phases are related to Ag^0^ and AgCl. SAED analysis of the electron-dense NPs reveals diffraction reflections corresponding to Ag, AgCl, and NaCl, as shown in Figure 6. The formation of natural nanoparticles could be described as erratic compared to that of artificial nanoparticles due to their polydispersity. The large number of precursors involved in the formation of natural nanoparticles, along with the fact that reactions can be incomplete or low yield, impacts the polydispersity and purity of the final product (Griffin et al., 2017). All particle populations synthesized were polydisperse, as indicated by PDI values lower than 1 (International Organization for Standardization, 2017); this implies a broad distribution of particle dimensions, potentially involving aggregation or the coexistence of multiple particle groups. According to the TEM images, a less electron-dense layer surrounds the particles, as shown in Figure 5c. ATR-FTIR analysis confirms the presence of organic molecules coating the purified nanoparticles (Figure 5d).

**FIGURE 7:**
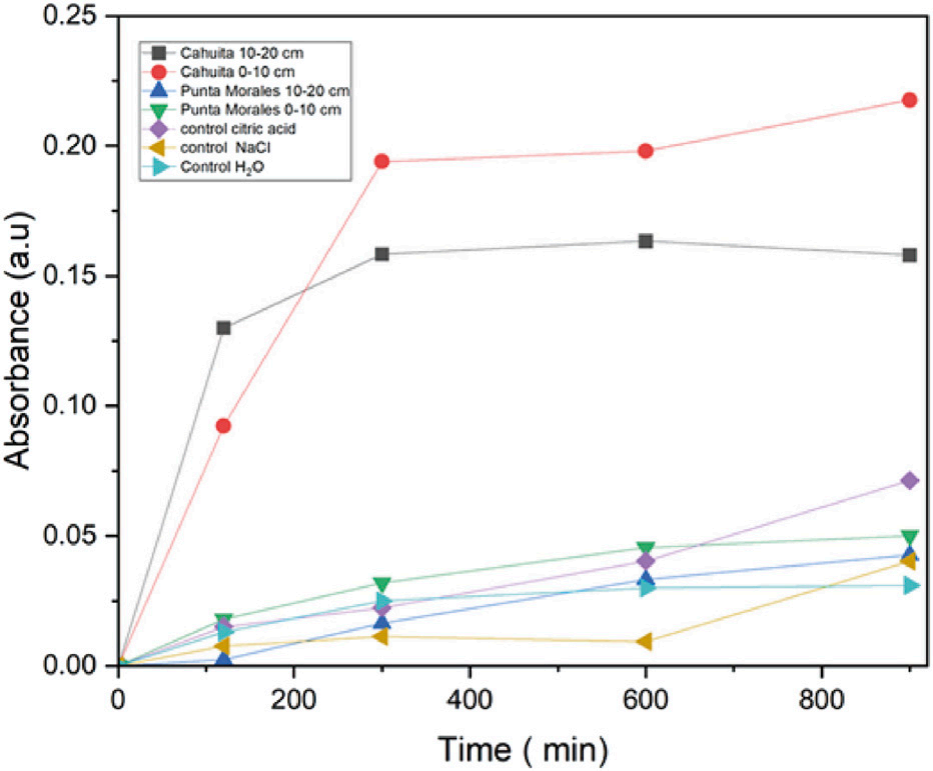
Variation in absorbance over time for the solution containing mangrove soil extracts and 0.1 M AgNO_3_, along with control solutions of AgNO_3_ with citric acid, AgNO_3_ with NaCl, and deionized H_2_O.

**FIGURE 8:**
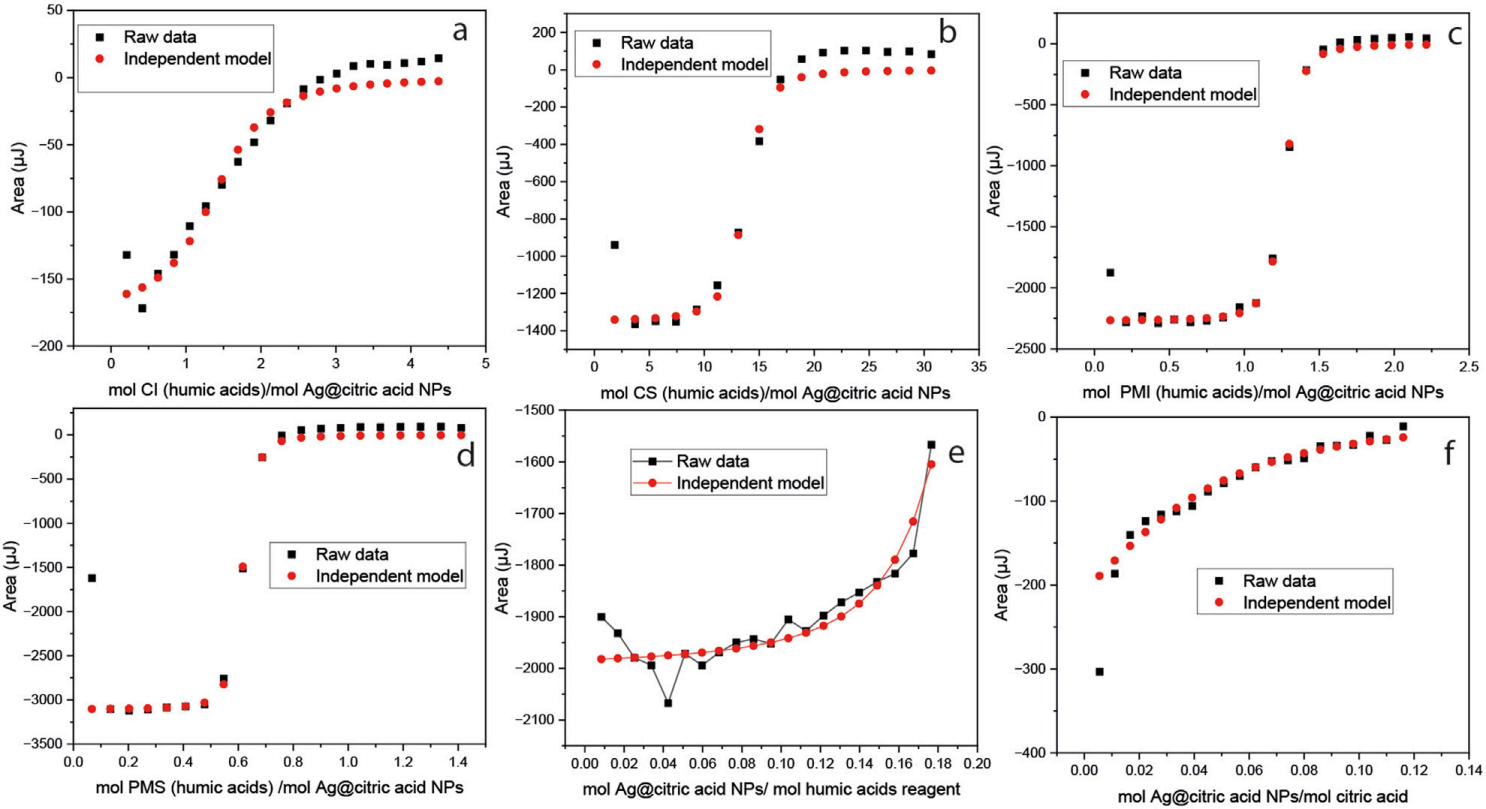
ITC results: enthalpy versus molar ratio and the modeled curves fitted with an independent model for **(a)** CI, **(b)** CS, PMS **(c)** PMI, and **(d)** PMS in the cell, titrated with AgNPs; **(e)** humic acids and **(f)** citric acid control in the syringe, with AgNPs in the cell.

To determine the rate of NPs formation using the mangrove extract, the absorbance of the solution was adjusted after different exposure times to sunlight; the trend is shown in Figure 7 (average values plotted). A control experiment was conducted to investigate the formation of Ag-NPs using citric acid, assuming that the organic matter was solely citric acid, to compare the effectiveness of the system in forming these substances with that of the mangrove extracts. Faster nanoparticle formation was obtained for the CI extracts. This could be correlated with the higher number of organic compounds and carbon- and nitrogen-containing species described above in Tables 3, 4. The CI catalyzed a faster formation of the Ag NPs in comparison with the same amount of mass of citric acid, and this can primarily be driven by affinity interactions (such as binding strength to a catalyst or substrate) rather than by the mobility of the reactant, which can be hindered by high molecular weight, as more mols were added to the reaction with the citric acid. To investigate the factors driving faster NP formation, ITC was used to determine the dissociation constant (Kd = Koff/Kon). Affinity comparisons between mangrove extracts were made using NP titrations, and humic acids were used as a model ligand (MW = 292 g/mol) to estimate concentrations in mM. Data were analyzed using the independent model, assuming multiple equivalent binding sites per NP (Figure 8; Table 6).

**TABLE 6:** ITC modeling results of the interaction of the mangrove extracts, humic acids, and citric acid with AgNPs synthesized.

Results	CI	CS	PMI	PMS	HA	AC
Kd (M)	1.17 × 10^−5^	4.4 × 10^−5^	9.31 × 10^−7^	3,351 × 10^−7^	1.1 × 10^−4^	3.63 × 10^−4^
n	1.50	11.4	1.214	0.547	0.15	0,0205
ΔH (kJ/mol)	−6.80	−4.49	−49.2	−112.65	−28.98	−18.2805
Ka (Mˉ^1^)	9.51 × 10^−4^	5.07 × 10^−4^	1.0745 × 10^6^	2,995 × 10^6^	61,666	2820.5
-TΔS (kJ/mol)	−21.5	−21.4	14.775	75.7	4.009	−1.381
ΔG (kJ/mol)	−28.3	−25.9	−34.425	−36.96	−24.97	−19.66
ΔS (J/mol·K)	72.1	71.8	−49.56	−253.9	−13.4555	4.64

The results suggest that extracts from soils in the Pacific area have a greater affinity for silver NPs than those from the Caribbean regions, as well as the humic acid control. However, the stoichiometry *n*1 represents the number of moles of Ag per mole of the material, indicating that, for example, in the case of CI, 1.5 mol of organic matter in humic acid equivalent interact with 1 mol of AgNPs in comparison to the PMS, influencing the reaction kinetics. Having more affinity for the NPs does not mean that a certain amount of extract can interact with a higher number of Ag NPs, as exemplified when comparing the n CI with PMS. The more positive value of −TΔS (which means a more negative ΔS) suggests that the entropy of the system decreases, indicating that it is becoming more ordered, such as when strong specific interactions like hydrogen bonding or electrostatic interactions dominate, as it is suggested for PMI and PMS (Callies and Hernández-Daranas, 2016).

**FIGURE 9:**
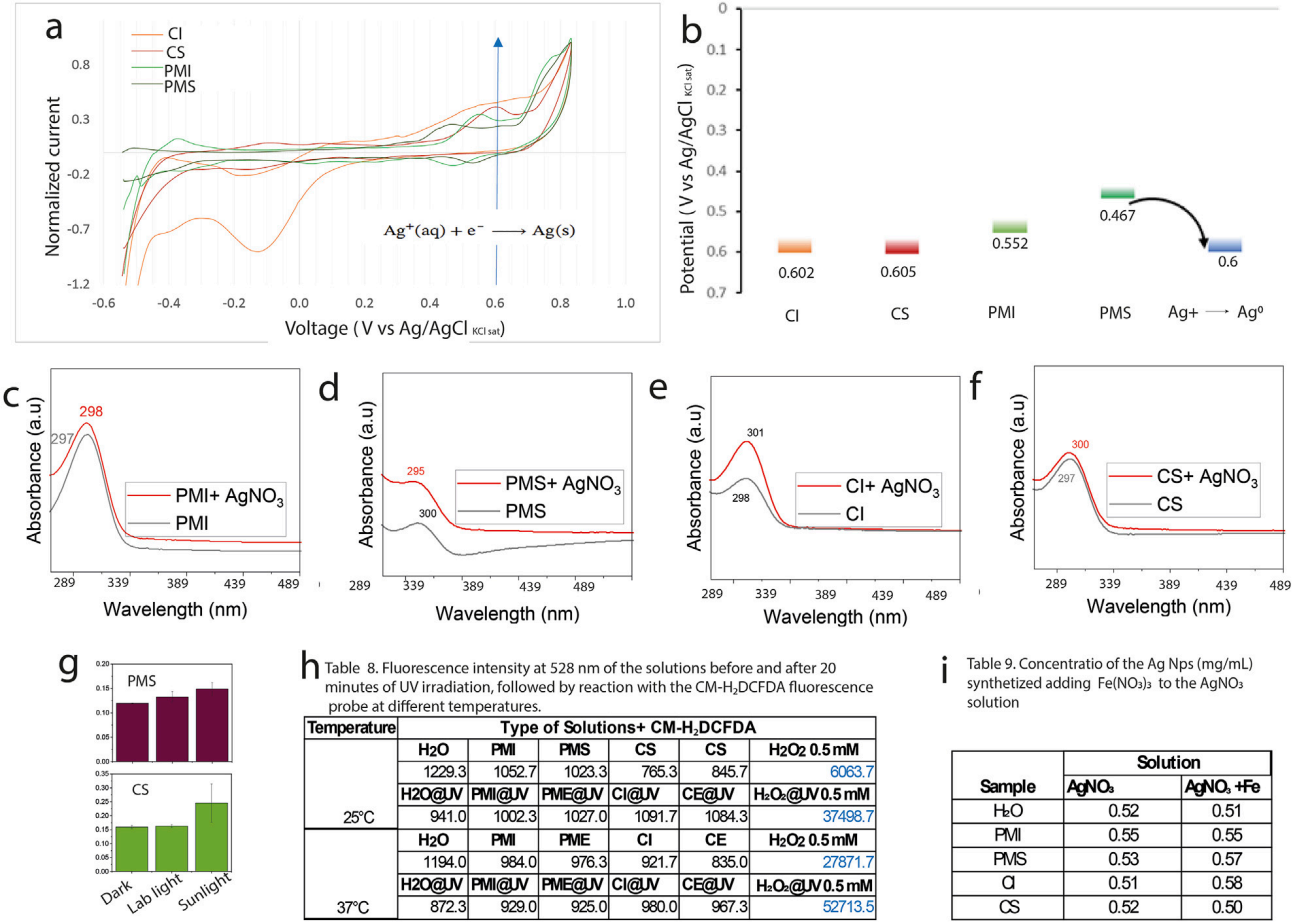
Mechanism of silver nanoparticle formation. **(a)** Cyclic voltammogram of the extracts (vs. Ag/AgCl). **(b)** Oxidation potentials relative to the Ag^+^/Ag^0^ couple. **(c–f)** UV-Vis absorbance of the extracts—**(c)** PMI, **(d)** PMS, **(e)** CI, and **(f)** CS—before and after mixing with AgNO_3_ in the dark, immediately prior to measurement. Final concentrations were 2 mg/mL of extract and 0.1 mg/mL AgNO_3_. **(g)** Absorbance at 15 min after nanoparticle formation under different lighting conditions (darkness, laboratory white light, and sunlight) for the PMS and CS extracts. **(i)** Concentration of AgNPs upon addition of Fe to the solution. **(j)** Table showing the average fluorescence intensity at 528 nm of CM-H_2_DCFDA after 30 min of incubation at 25°C or 37°C.

To gain insight into the molecular mechanisms involved in nanoparticle formation, five experiments were conducted. Figure 9a shows the cyclic voltammogram of mangrove extracts (vs. Ag/AgCl). The lower the oxidation potential of an organic compound, the stronger its reducing character. The reduction potential of the Ag^+^/Ag^0^ couple (from AgNO_3_), referenced against Ag/AgCl (saturated KCl), is approximately +0.602 V. Among the organic species analyzed, the lowest oxidation potential is 0.460 V (Figure 9b). This suggests that species such as PMI and PS can reduce Ag^+^, with a positive cell potential indicating spontaneous electron transfer. In contrast, CI has an oxidation potential close to that of Ag^+^/Ag^0^ and may only marginally drive the reduction. CS, with a slightly higher oxidation potential, is likely to lead to a non-spontaneous reaction under the same conditions. However, solar irradiation or mild thermal activation can significantly influence these borderline cases. Another possibility is that in the complex formed by ionic silver and the organic matter, charge transfers occur from the ligand to the metal, causing the direct reduction of silver (Hou, Stuart, Howes and Zepp, 2013). Figures 9c–f. Shows the peak shifting in PMI, CI, and CS to higher wavelengths, and PMS to lower wavelengths. This shifting of the peaks suggests a possible complexation of the silver ions by the organic matter. Another mechanism that could be involved is photoinduced reactions, where a species is reduced by agents such as sugars or organic matter when the system is exposed to natural light, as in the case of mangroves (Adegboyega et al., 2016). Photonic energy can reduce activation barriers, excite electrons in photoactive species, and enhance electron transfer kinetics, thereby enabling reactions to proceed even when redox potentials are closely matched or slightly unfavorable under dark conditions. When we compared the increase in absorbance under dark conditions, standard laboratory lighting, and sunlight exposure, we observed that light enhanced the kinetics of nanoparticle formation. This suggests that photonic energy may play a role in lowering activation barriers, Figure 9g. In this case, the system’s exposure to UV and visible light enables different pathways for the reduction of Ag^+^. One possible mechanism is that photolysis of the organic matter leads to the formation of phenolic-derived radicals, which react with dissolved oxygen to generate superoxide radical anions (O_2_•^−^), capable of reducing silver ions to metallic silver (Yin et al., 2012). Figure 9h shows a table summarizing the fluorescence intensity results obtained from the reaction of the probe with the mangrove extract after 20 min of UV-vis irradiation at 254 nm, compared with non-irradiated control samples. An ANOVA test using Milli-Q water as a control revealed no significant difference in fluorescence intensity between the mangrove extract and the control. Only the positive samples containing 0.5 mM H_2_O_2_, with or without UV-vis irradiation, showed a significant increase in fluorescence intensity relative to the control. Notably, higher fluorescence intensity was observed in the H_2_O_2_ samples after UV exposure. The incubation with the fluorescent probe was conducted at two different temperatures to accelerate the reaction kinetics, but similar results were obtained in both cases. These results suggest that the ROS formation is not a significant mechanism for nanoparticle formation. The presence of other species in the system may catalyze or interfere with the reaction; for example, the natural presence of Cl^−^ in water generates AgCl(s) (as commented above in the EDX analysis) and acts as a substrate in the autocatalysis of the reduction reaction. At the same time, Na^+^ ions may compete for binding sites in the organic matter. The presence of iron species may facilitate the reaction by forming higher-charge complexes with the organic matter without directly competing with Ag^+^. Additionally, Fe^2+^/Fe^3+^ ions can act as charge carriers that promote the reduction of silver. Figure 9i presents a table comparing the average concentration of synthesized nanoparticles obtained with the addition of Fe(NO_3_)_3_ versus AgNO_3_ alone. A one-way ANOVA test indicates that the concentrations are significantly different, with nanoparticle production increasing for both PMS and CI upon the addition of iron, but not for all extracts.

Another factor influencing these reactions is the presence of nitrogen and sulfur-containing species in the soil (Sharma et al., 2015). Further studies with more detailed chemical characterization of the involved compounds are necessary to determine the role of specific molecules. Microorganism-mediated nanoparticle formation is perhaps one of the most complex processes in nature (Bakshi et al., 2015; Griffin et al., 2017; Sharma et al., 2015). However, since the mangrove extracts were filtered, this mechanism is relevant in this study, except for the possible involvement of extracellular molecules produced by microorganisms. Nonetheless, this could represent another relevant mechanism for nanoparticle formation in the mangrove ecosystem.

### 3.2 Minimal inhibitory concentration

The antimicrobial activity of nanoparticles obtained from mangrove species has been proven on numerous occasions (Willian et al., 2020; Alsareii et al., 2022; Soni et al., 2024). These same genera are distributed in Costa Rica (Silva-Benavides, 2009). Considering the biodegradation dynamics that occur in mangrove forests, where around 30% of the sedimentary carbon corresponds to microorganisms (Alongi, 2005; Carugati et al., 2018), the high concentration of tannins, polyphenols, cellulose and lignin favor a slower decomposition (Booth et al., 2023; Wang et al., 2024). Some of these compounds are part of the sediment in mangrove forests, interacting with metals in a recycling dynamic (Lang et al., 2024). These compounds also favor the formation of metallic nanoparticles (Gangwar et al., 2021). The antimicrobial activity of nanoparticles is significantly influenced by factors such as size, surface charge, shape, and the material’s nature (Dizaj et al., 2014). Smaller nanoparticles typically exhibit higher antimicrobial activity than larger ones (Agnihotri et al., 2014). For example, Suchomel et al. (2015) synthesized nanoparticles ranging from 60 to 120 nm and reported bacterial inhibition at a concentration of 5.1 μg/mL. In contrast, other studies reported minimum inhibitory concentrations (MICs) of 9.7 × 10^ (−7) and 0.8 μg/mL for nanoparticles sized at 8.8 nm and 28 nm, respectively (Abbaszadegan et al., 2015; Panáček et al., 2016). The antimicrobial effect of silver nanoparticles is well-documented, with mechanisms linked to the generation of reactive oxygen species and DNA damage (Sharma et al., 2015; Levard et al., 2013). All the NPs synthesized are polydisperse, as suggested by the DLS and TEM analysis (Supplementary Figure S7); however, the main population has a diameter of less than 100 nm. Organic coatings can substantially modify the behavior of nanoparticles in biological and environmental systems. Different amounts of organic matter can inhibit aggregation through steric hindrance and electrostatic repulsion, which may influence the biological response (Li et al., 2020; Doane et al., 2012; Vindedahl et al., 2016). For the synthesized particles, the amount of weight loss of the organic matter surrounding the particles, as measured by TGA, was similar indicating that the results obtained are not influenced by the amount of matter but for the type of organic matter (Supplementary Figure S3). *Z*eta potential analyses revealed significant differences in surface charge among the synthesized nanoparticles (Supplementary Figure S8). These variations point to distinct surface coatings and differences in aggregation behavior, which are known to influence nanoparticle interactions and biological activity. Organic shells or capping agents, such as those derived from plant extracts, are well-documented modulators of nanoparticle toxicity (Bellingeri et al., 2024). Zeta potential values varied between nanoparticle formulations, further suggesting that different molecular compositions or orientations of the organic coatings could influence thier activity (Supplementary Figure S7). While some nanoparticles were more strongly negatively charged than others, PMS and CS exhibited zeta potential values below −20 mV or higher than 20 mV, indicating good colloidal stability in aqueous media. These differences reinforce the conclusion that variations in the organic shell influence not only nanoparticle stability but also biological interactions and antimicrobial activity due to its net charge. These coatings also impact nanoparticle reactivity by influencing surface adsorption, phase transformation dynamics, and toxicity profiles. However, the Gram-positive bacteria had a lower MIC when treated with the CI and PMI Ag NPs, suggesting the electrostatic interactions are not the main driving force of antimicrobial activity. Khoshnamvand et al. (2020) found that organic coatings reduced the toxicity of silver nanoparticles to *Chlorella vulgaris* by decreasing their bioavailability and subsequent cellular damage. However, organic coatings do not uniformly reduce toxicity: in some cases, as with titanium dioxide nanoparticles, the presence of humic acids can increase toxicity (Tang et al., 2014), likely due to enhanced transport or cellular uptake.

**FIGURE 10:**
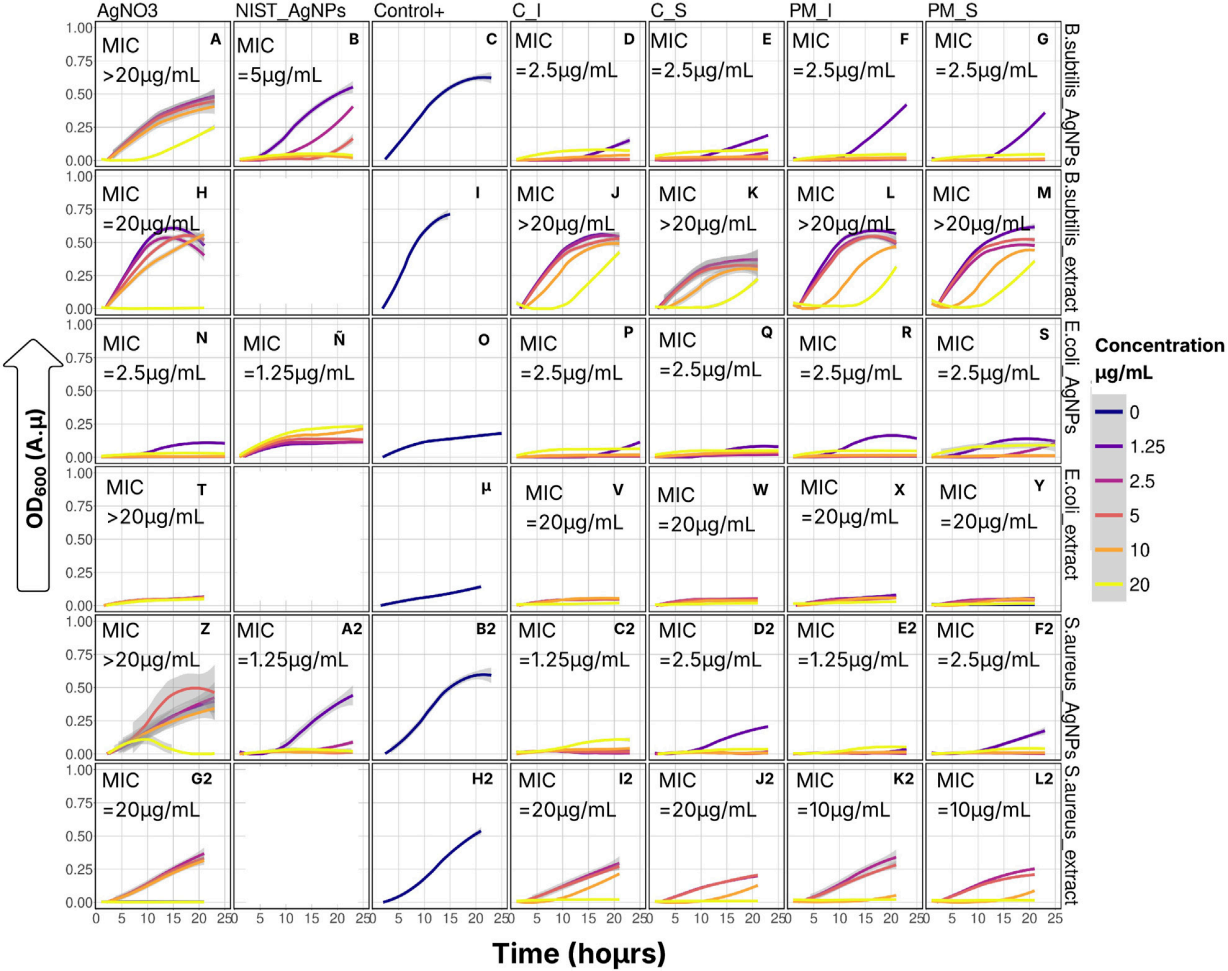
Bacterial growth with time of *E. coli*, S. Subtilis, and *S. Aureus* 1 X10^−5^ CFU/mL and different NPs concentrations.

To determine whether the extract alone contributed to the observed antimicrobial activity, control assays were conducted using the same concentrations of extract (without silver) as those used in the nanoparticle formulations. These showed inhibitory effects on bacterial growth only for the highest concentration (Figure 10), indicating that the antimicrobial action in, *B. subtilis* (resistant spores, Gram positive), S. aureus (Gram positive) and *E. coli* (Gram negative) arises mainly from a synergistic interaction between silver and the organic coating, rather than from the extract alone.

Given the complex interactions between nanoparticles and biological systems, a linear relationship between nanoparticle dose and toxicity cannot be assumed. Environmental factors and microbial metabolic products contribute to the formation of a nanoparticle–biomolecule corona, which can significantly alter biological interactions and antimicrobial efficacy (Cedervall et al., 2007; Kamat and Kumari, 2023).

To better understand the temporal dynamics of microbial growth in response to the treatments, a generalized additive mixed model (GAM, Supplementary Figure S8) was fitted using the formula: OD_600_ ∼ Treatment × Bacteria + s (Time, k = 4, by = Concentration). This model explained 62.4% of the variance in the optical density data from extract-based assays (adjusted R^2^ = 0.624, p < 0.01, n = 5,334) and 53% of the variance in nanoparticle-based assays (adjusted R^2^ = 0.529, p < 0.01, n = 1,440) (For comparisons details see Supplementary Figures S8–S10).

For the extract-based assays, neither sampling location (Cahuita vs. Punta Morales) nor depth (surface vs. internal strata) produced significant differences in antimicrobial activity across the three tested bacterial species (Holm-adjusted p > 0.01). However, growth inhibition patterns varied significantly with bacterial species and extract concentration (Holm-adjusted p < 0.01). Among the tested strains, *E. coli* exhibited the highest sensitivity to the extracts, followed by *S. aureus*, while *B. subtilis* was the most resistant. Notably, only the highest extract concentration (20 μg/mL) was effective in suppressing *B. subtilis* growth within the first 10 h. In contrast, *E. coli* growth was suppressed at all tested concentrations, although 20 μg/mL was required to reduce the OD_600_ to near zero. For *S. aureus*, a concentration of 10 μg/mL was sufficient to achieve similar inhibition during the early stages of incubation.

A similar species-specific inhibition trend was observed in nanoparticle treatments. *B. subtilis* again showed the least sensitivity and did not respond significantly to variations in nanoparticle origin or concentration. However, control silver nanoparticles (commercially synthesized; NIST_AgNPs) elicited significant, concentration-dependent growth inhibition in *E. coli, S. aureus, and B. subtilis* (*p* < 0.001). Interestingly, for *E. coli*, the highest nanoparticle concentration did not yield maximal inhibition. Instead, OD_600_ values increased slightly relative to lower concentrations, possibly reflecting a hormetic response or an aggregation-related reduction in antimicrobial efficacy.

Further comparisons using the Scheirer–Ray–Hare test with Dunn’s *post hoc* contrasts confirmed that there were no statistically significant differences in OD_600_ values between extracts or nanoparticles from CI, CS, PMS and PMI across all three bacterial species. Similarly, comparisons between CI and PMI for extract treatments, and between CS and PMS for nanoparticle treatments, yielded no significant differences. For *E. coli*, extract treatments from CS and CI produced equivalent levels of inhibition (Supplementary Figure S9).

The elevated OD_600_ values observed in assays using NIST silver nanoparticles are likely attributable to their polyvinylpyrrolidone (PVP) coating, which is known to reduce silver ion release and subsequent antimicrobial activity (Rodrigues et al., 2024). In *E. coli*, sub-inhibitory concentrations of AgNPs can trigger overexpression of flagellin, which facilitates nanoparticle aggregation and precipitation, thereby decreasing bioavailability. *S. aureus* responds similarly by increasing biofilm production, which entraps nanoparticles and reduces their antimicrobial efficacy (Hochvaldová et al., 2024). *B. subtilis* may employ comparable mechanisms (Arnaouteli et al., 2021), explaining the increased OD_600_ values at higher nanoparticle concentrations due to reduced antibacterial activity.

Interestingly, *B. subtilis* growth appeared to be stimulated by extracts from PMI and PMS, suggesting a growth-promoting role for specific ions that are present in the soil extracts. Magnesium, an essential cofactor for numerous cellular processes including division, and iron, which is tightly regulated by metalloregulatory proteins (e.g., Fur, MntR, PerR), are key contributors to this effect (Guo and Herman, 2023; Helmann, 2014; Steingard et al., 2023). Other trace elements such as zinc, manganese, and calcium, alongside organic matter present in the extracts, may further support microbial homeostasis and resilience (Martin et al., 2015; Diekman et al., 2024). The apparent reduction in inhibitory effects at specific extract concentrations may thus be attributed to enhanced metabolic support or increased availability of trace nutrients.

Overall, the environmental implications of AgNP exposure are complex and highly context-dependent. AgNPs have demonstrated the capacity to disrupt microbial metabolism and community structure, particularly in soils, where factors such as particle size, surface charge, and chemical coatings influence their toxicity (Ottoni et al., 2020). Although valued for their antimicrobial properties, the introduction of AgNPs into sensitive environments—such as mangroves, where microbial communities are essential for nitrogen cycling—may pose significant ecological risks (Das et al., 2013; Mubaraq et al., 2024). While smaller, uncoated nanoparticles are generally more toxic, natural transformations such as sulfidation and interactions with organic matter can attenuate these effects, potentially mitigating their long-term environmental impact (McGee, 2020). Our studies suggest that NPs coated with organic matter, in general, are less toxic than commercial ones coated with PVP. Further studies are needed to understand better how surrounding organic matter interacts with particles and influences nanoparticle dissolution. Additionally, comparisons with other systems, such as Ag films coated or uncoated with organic matter, to exclude the influence in NPs sizes and aggregations. In this study, we were unable to compare Ag NPs without coatings, this process introduces additional challenges, including precipitation and agglomeration, which alter the potential interactions of the NPs with the bacteria. On the positive side, biogenically formed AgNPs offer eco-compatible tools for pollution control, including the degradation of organic pollutants (e.g., dyes, pharmaceuticals), antimicrobial activity for contaminated water or sediments, and heavy metal removal via sorption and reduction mechanisms. This opens a sustainable pathway for nanoparticle-mediated remediation, particularly in coastal or estuarine zones affected by urban, agricultural, or industrial runoff. Further studies are necessary to determine whether these formed nanoparticles from metal-containing waters can aid in bioremediation.

## 4 Conclusions

Ecosystems such as mangroves have gained increasing attention for their ability to act as natural reducing agents, primarily through humic and fulvic substances in the soil, as well as extracellular molecules produced by microorganisms. This study aimed to assess the potential of Costa Rican mangroves in forming nanostructured materials and to compare the differences between Pacific and Caribbean mangrove ecosystems. These differences may offer valuable insights into their capacity to absorb heavy metals and produce nanoparticles. For the first time, nanoparticles associated with silicates and iron oxides were identified within the mangrove ecosystem. Nanoparticle synthesis occurred within a short time frame under sunlight exposure and high salinity, without the need for another external energy source. Extracts from the Punta Morales mangroves showed a stronger affinity for silver nanoparticles, while those from Cahuita facilitated quicker nanoparticle formation. These contrasting behaviors could be attributed to a higher number of binding sites in the extracts, which influence the nanoparticle synthesis process. The synthesized NPs have lower antimicrobial activity that commercial PVP-coated Ag NPs. The results revealed notable distinctions between the two regions, suggesting that the Cahuita mangroves may exhibit greater resilience to metal contamination—possibly due to their enhanced ability to form nanoparticles. However, the rapid formation of these particles may also have implications for the ecosystem’s microbial communities.

## Data Availability

The data presented in the study are deposited in the zenodo. org respository, accesion DOI: 10.5281/zenodo.16755567.

## References

[B1] Abbaszadegan A. Ghahramani Y. Gholami A. Hemmateenejad B. Dorostkar S. Nabavizadeh M. (2015). The effect of charge at the surface of silver nanoparticles on antimicrobial activity against Gram-positive and Gram-negative bacteria: a preliminary study. J. Nanomater. 2015, 1–8. 10.1155/2015/720654

[B2] Adegboyega N. F. Sharma V. K. Cizmas L. Sayes C. M. (2016). UV light induces Ag nanoparticle formation: roles of natural organic matter, iron, and oxygen. Environ. Chem. Lett. 14 (3), 353–357. 10.1007/s10311-016-0577-z

[B3] Agnihotri S. Mukherji S. Mukherji S. (2014). Size-controlled silver nanoparticles synthesized over the range 5–100 nm using the same protocol and their antibacterial efficacy. RSC Adv. 4, 3974–3983. 10.1039/C3RA44507K

[B4] Alongi D. M. (2005). Mangrove-microbe-soil relations. Coast. Estuar. Stud. 60, 85–103. 10.1029/CE060p0085

[B5] Alsareii S. A. Manaa Alamri A. AlAsmari M. Y. Bawahab M. A. Mahnashi M. H. Shaikh I. A. (2022). Synthesis and characterization of silver nanoparticles from *Rhizophora apiculata* and studies on their wound healing, antioxidant, anti-inflammatory, and cytotoxic activity. Molecules 27 (19), 6306. 10.3390/molecules27196306 36234841 PMC9571849

[B6] Arnaouteli S. Bamford N. C. Stanley-Wall N. R. Kovács Á. T. (2021). *Bacillus subtilis* biofilm formation and social interactions. Nat. Rev. Microbiol. 19 (9), 600–614. 10.1038/s41579-021-00540-9 33824496

[B7] Augusthy S. Nizam A. Kumar A. (2024). The diversity, drivers, consequences and management of plant invasions in the mangrove ecosystems. Sci. Total Environ. 945, 173851. 10.1016/j.scitotenv.2024.173851 38871312

[B8] Bakshi S. He Z. L. Harris W. G. (2015). Natural nanoparticles: implications for environment and human health. Crit. Rev. Environ. Sci. Technol. 45 (8), 861–904. 10.1080/10643389.2014.921975

[B9] Bellingeri A. Bono N. Venditti I. Bertelà F. Burratti L. Faleri C. (2024). Capping drives the behavior, dissolution and (eco)toxicity of silver nanoparticles towards microorganisms and Mammalian cells. Environ. Sci. Nano 11 (5), 2049–2060. 10.1039/D4EN00063C

[B10] Booth J. M. Fusi M. Marasco R. Daffonchio D. (2023). The microbial landscape in bioturbated mangrove sediment: a resource for promoting nature-based solutions for mangroves. Microb. Biotechnol. 16 (8), 1584–1602. 10.1111/1751-7915.14273 37209285 PMC10364319

[B11] Bunting P. Rosenqvist A. Hilarides L. Lucas R. M. Thomas N. Tadono T. (2022). Global mangrove extent change 1996–2020: global mangrove watch version 3.0. Remote Sens. 14 (15), 3657. 10.3390/rs14153657

[B12] Callies O. Hernández-Daranas A. (2016). Application of isothermal titration calorimetry as a tool to study natural product interactions. Nat. Product. Rep. 33 (7), 881–904. 10.1039/C5NP00094G 27186603

[B13] Carugati L. Gatto B. Rastelli E. Lo Martire M. Coral C. Greco S. (2018). Impact of mangrove forests degradation on biodiversity and ecosystem functioning. Sci. Rep. 8 (1), 13298. 10.1038/s41598-018-31683-0 30185918 PMC6125342

[B14] Cedervall T. Lynch I. Lindman S. Berggård T. Thulin E. Nilsson H. (2007). Understanding the nanoparticle–protein Corona using methods to quantify exchange rates and affinities of proteins for nanoparticles. Proc. Natl. Acad. Sci. 104 (7), 2050–2055. 10.1073/pnas.0608582104 17267609 PMC1892985

[B15] Choudhary B. Dhar V. Pawase A. S. (2024). Blue carbon and the role of mangroves in carbon sequestration: its mechanisms, estimation, human impacts and conservation strategies for economic incentives. J. Sea Res. 199, 102504. 10.1016/j.seares.2024.102504

[B16] Das B. K. Chakraborty H. J. Kumar V. Rout A. K. Patra B. Das S. K. (2024). Comparative metagenomic analysis from sundarbans ecosystems advances our understanding of microbial communities and their functional roles. Sci. Rep. 14 (1), 16218. 10.1038/s41598-024-67240-1 39003345 PMC11246455

[B17] Das D. Ghosh S. Banerjee S. (2022). A review of metal resistance mechanisms by mangrove bacteria. Res. J. Biotechnol. 17 (3), 209–215. 10.25303/1703rjbt209215

[B18] Das S. Ganguly D. Maiti T. K. Mukherjee A. Jana T. K. De T. K. (2013). A depth-wise diversity of free-living N2 fixing and nitrifying bacteria and its seasonal variation with nitrogen-containing nutrients in the mangrove sediments of sundarban, WB, India. Open J. Mar. Sci. 3 (2), 112–119. 10.4236/ojms.2013.32012

[B19] Da Silva R. R. Lucena G. N. Machado Â. F. de Freitas G. A. Matos A. T. Abrahão W. A. P. (2018). Spectroscopic and elementary characterization of humic substances in organic substrates. Comun. Sci. 9 (2), 264–274. 10.14295/cs.v9i2.2734

[B20] De Melo B. A. G. Motta F. L. Santana M. H. A. (2016). Humic acids: structural properties and multiple functionalities for novel technological developments. Mater. Sci. Eng. C 62, 967–974. 10.1016/j.msec.2015.12.001 26952503

[B21] Diekman C. M. Cook C. Strawn L. K. Danyluk M. D. (2024). Factors associated with the prevalence of *salmonella*, generic *Escherichia coli*, and coliforms in Florida’s agricultural soils. J. Food Prot. 87 (5), 100265. 10.1016/j.jfp.2024.100265 38492643

[B22] Ding J. Liu F. Zeng J. Li Y. Yang Y. Liu X. (2025). Depth heterogeneity of lignin-degrading microbiome and organic carbon processing in mangrove sediments. npj Biofilms Microbiomes 11, 5. 10.1038/s41522-024-00638-x 39762227 PMC11704145

[B23] Dizaj S. M. Lotfipour F. Barzegar-Jalali M. Zarrintan M. H. Adibkia K. (2014). Antimicrobial activity of the metals and metal oxide nanoparticles. Mater. Sci. Eng. C 44, 278–284. 10.1016/j.msec.2014.08.031 25280707

[B24] Doane T. L. Chuang C.-H. Hill R. J. Burda C. (2012). Nanoparticle ζ-potentials. Accounts Chem. Res. 45 (3), 317–326. 10.1021/ar200113c 22074988

[B25] Furusawa G. (2019). Invited review: biodiversity of plant polysaccharide-degrading bacteria in mangrove ecosystem. Trop. Life Sci. Res. 30 (3), 157–172. 10.21315/tlsr2019.30.3.11

[B26] Gangwar C. Yaseen B. Kumar I. Singh N. K. Naik R. M. (2021). Growth kinetic study of tannic acid mediated monodispersed silver nanoparticles synthesized by chemical reduction method and its characterization. ACS Omega 6 (34), 22344–22356. 10.1021/acsomega.1c03100 34497923 PMC8412910

[B27] Glover R. D. Miller J. M. Hutchison J. E. (2011). Generation of metal nanoparticles from silver and copper objects: nanoparticle dynamics on surfaces and potential sources of nanoparticles in the environment. ACS Nano 5 (11), 8950–8957. 10.1021/nn2031319 21985489

[B28] Griffin S. Masood M. I. Nasim M. J. Sarfraz M. Ebokaiwe A. P. Schäfer K. H. (2017). Natural nanoparticles: a particular matter inspired by nature. Antioxidants 7 (1), 3. 10.3390/antiox7010003 29286304 PMC5789313

[B29] Guo T. Herman J. K. (2023). Magnesium modulates *Bacillus subtilis* cell division frequency. J. Bacteriol. 205 (1), e0037522–e0037522. 10.1128/jb.00375-22 36515540 PMC9879117

[B30] Gupta S. Kulkarni M. G. White J. F. Stirk W. A. Papenfus H. B. Doležal K. (2021). “Categories of various plant biostimulants—Mode of application and shelf-life,” in Biostimulants for crops from seed germination to plant development (Elsevier), 1–60. 10.1016/B978-0-12-823048-0.00018-6

[B31] Helmann J. D. (2014). Specificity of metal sensing: iron and manganese homeostasis in *Bacillus subtilis* . J. Biol. Chem. 289 (41), 28112–28120. 10.1074/jbc.R114.587071 25160631 PMC4192466

[B32] Hochvaldová L. Panáček D. Válková L. Večeřová R. Kolář M. Prucek R. (2024). *E. coli* and *S. aureus* resist silver nanoparticles *via* an identical mechanism, but through different pathways. Commun. Biol. 7 (1), 1552. 10.1038/s42003-024-07266-3 39572718 PMC11582817

[B33] Hou W. C. Stuart B. Howes R. Zepp R. G. (2013). Sunlight-driven reduction of silver ions by natural organic matter: formation and transformation of silver nanoparticles. Environ. Sci. and Technol. 47 (14), 7713–7721. 10.1021/es400802w 23731169

[B34] International Organization for Standardization (2017). Particle size analysis—dynamic light scattering (DLS). ISO 22412:2017. International Organization for Standardization, February, 2017. Available online at: https://www.iso.org/standard/65410.html.

[B35] Kadeřábková N. Mahmood A. J. S. Mavridou D. A. I. (2024). Antibiotic susceptibility testing using minimum inhibitory concentration (MIC) assays. npj Antimicrob. Resist. 2 (1), 37. 10.1038/s44259-024-00051-6 39843555 PMC11721449

[B36] Kaiprath S. S. Singh R. Lekhak U. M. (2024). Naturally occurring nanoparticles (NONPs): a review. Next Sustain. 3, 100037. 10.1016/j.nxsust.2024.100037

[B37] Kamat S. Kumari M. (2023). Emergence of microbial resistance against nanoparticles: mechanisms and strategies. Front. Microbiol. 14, 1102615. 10.3389/fmicb.2023.1102615 36778867 PMC9909277

[B38] Kathiresan K. Alikunhi N. M. Nabikhan A. (2012). *In vitro* synthesis of antimicrobial silver nanoparticles by mangroves, saltmarshes and plants of coastal origin. Int. J. Biomed. Nanosci. Nanotechnol. 2 (3/4), 284. 10.1504/IJBNN.2012.051222

[B39] Kathiresan K. Manivannan S. Nabeel M. A. Dhivya B. (2009). Studies on silver nanoparticles synthesized by a marine fungus, *penicillium Fellutanum* isolated from coastal mangrove sediment. Colloids Surfaces B Biointerfaces 71 (1), 133–137. 10.1016/j.colsurfb.2009.01.016 19269142

[B40] Khoshnamvand M. Ashtiani S. Chen Y. Liu J. (2020). Impacts of organic matter on the toxicity of biosynthesized silver nanoparticles to green microalgae *Chlorella vulgaris* . Environ. Res. 185, 109433. 10.1016/j.envres.2020.109433 32247152

[B41] Kida M. Tomotsune M. Iimura Y. Kinjo K. Ohtsuka T. Fujitake N. (2017). High salinity leads to accumulation of soil organic carbon in mangrove soil. Chemosphere 177, 51–55. 10.1016/j.chemosphere.2017.02.074 28282623

[B42] Klučáková M. Pavlíková M. (2017). Lignitic humic acids as environmentally-friendly adsorbent for heavy metals. J. Chem. 2017, 7169019. 10.1155/2017/7169019

[B43] Krishnamurthy P. Jyothi-Prakash P. A. Qin L. He J. Lin Q. Loh C. S. (2014). Role of root hydrophobic barriers in salt exclusion of a mangrove plant *Avicennia officinalis* . Plant, Cell and Environ. 37 (7), 1656–1671. 10.1111/pce.12272 24417377

[B44] Kuhn M. (2008). Building predictive models in R using the caret package. J. Stat. Softw. 28 (5). 10.18637/jss.v028.i05

[B45] Lang T. Ke X. Wei J. Hussain M. Li M. Gao C. (2024). Dynamics of tannin variations in mangrove leaf litter decomposition and their effects on environmental nitrogen and microbial activity. Sci. Total Environ. 908, 168150. 10.1016/j.scitotenv.2023.168150 37918719

[B46] Lee S. Y. Primavera J. H. Dahdouh-Guebas F. McKee K. Bosire J. O. Cannicci S. (2014). Ecological role and services of tropical mangrove ecosystems: a reassessment. Glob. Ecol. Biogeogr. 23 (7), 726–743. 10.1111/geb.12155

[B47] Levard C. Mitra S. Yang T. Jew A. D. Badireddy A. R. Lowry G. V. (2013). Effect of chloride on the dissolution rate of silver nanoparticles and toxicity to *E. coli* . Environ. Sci. and Technol. 47 (11), 5738–5745. 10.1021/es400396f 23641814

[B48] Li P. Li X. Bai J. Meng Y. Diao X. Pan K. (2022). Effects of land use on the heavy metal pollution in mangrove sediments: study on a whole island scale in Hainan, China. Sci. Total Environ. 824, 153856. 10.1016/j.scitotenv.2022.153856 35176367

[B49] Li Z. Shakiba S. Deng N. Chen J. Louie S. M. Hu Y. (2020). Natural organic matter (NOM) imparts molecular-weight-dependent steric stabilization or electrostatic destabilization to ferrihydrite nanoparticles. Environ. Sci. and Technol. 54 (11), 6761–6770. 10.1021/acs.est.0c01189 32250111

[B50] Mangiafico S. S. (2025). Rcompanion: functions to support extension education program evaluation. New Brunswick, NJ: Rutgers Cooperative Extension. Available online at: https://CRAN.R-project.org/package=rcompanion.

[B86] Martin J. E. Waters L. S. Storz G. Imlay J. A. (2015). The* Escherichia coli* small protein mnts and exporter mntp optimize the intracellular concentration of manganese. PLOS Genetics 11 (3), e1004977. 10.1371/journal.pgen.1004977 25774656 PMC4361602

[B85] Mathew J. Gopinath A. Vareed R. A. (2021). Spectroscopic characterization of humic substances isolated from tropical mangrove sediments. Arab. J. Geosci. 14 (8), 668. 10.1007/s12517-021-06968-w

[B87] McGee C. F. (2020). The effects of silver nanoparticles on the microbial nitrogen cycle: A review of the known risks. Environ. Sci. Pollut. Res. Int. 27 (25), 31061–31073. 10.1007/s11356-020-09548-9 32514926

[B84] Mo S. He S. Sang Y. Li J. Kashif M. Zhang Z. (2023). Integration of microbial transformation mechanism of polyphosphate accumulation and sulfur cycle in subtropical marine mangrove ecosystems with spartina alterniflora invasion. Microb. Ecol. 85 (2), 478–494. 10.1007/s00248-022-01979-w 35157108

[B51] Mubaraq A. Sembiring M. Widiastuti E. Fachrial E. Utomo B. Turjaman M. (2024). Diversity and distribution of nitrifying bacteria play an important role in the nitrogen cycle in mangrove sediments. Glob. J. Environ. Sci. Manag. 10 (4). 10.22034/gjesm.2024.04.39

[B52] Narendran R. Kathiresan K. (2016). Antimicrobial activity of crude extracts from mangrove-derived *trichoderma* species against human and fish pathogens. Biocatal. Agric. Biotechnol. 6, 189–194. 10.1016/j.bcab.2016.03.003

[B53] Ogle D. H. Doll J. C. Wheeler A. P. Dinno A. (2025). Data from: FSA: simple fisheries stock assessment methods. The R Foundation. 10.32614/cran.package.fsa

[B54] Ottoni C. A. Lima Neto M. C. Léo P. Ortolan B. D. Barbieri E. De Souza A. O. (2020). Environmental impact of biogenic silver nanoparticles in soil and aquatic organisms. Chemosphere 239, 124698. 10.1016/j.chemosphere.2019.124698 31493753

[B55] Palit K. Rath S. Chatterjee S. Das S. (2022). Microbial diversity and ecological interactions of microorganisms in the mangrove ecosystem: threats, vulnerability, and adaptations. Environ. Sci. Pollut. Res. 29 (22), 32467–32512. 10.1007/s11356-022-19048-7 35182344

[B56] Panáček A. Smékalová M. Večeřová R. Bogdanová K. Röderová M. Kolář M. (2016). Silver nanoparticles strongly enhance and restore bactericidal activity of inactive antibiotics against multiresistant enterobacteriaceae. Colloids Surfaces B Biointerfaces 142, 392–399. 10.1016/j.colsurfb.2016.03.007 26970828

[B57] Parisi M. Verrillo M. Luciano M. A. Caiazzo G. Quaranta M. Scognamiglio F. (2023). Use of natural agents and agrifood wastes for the treatment of skin photoaging. Plants 12 (4), 840. 10.3390/plants12040840 36840187 PMC9966275

[B58] Patra J. K. Mishra R. R. Thatoi H. (2020). Biotechnological utilization of mangrove resources. Elsevier Science and Technology.

[B59] Pumijumnong N. Uppadit B. (2013). Accumulation of heavy metals in mangrove sediments of Chumphon province, Thailand. Appl. Environ. Res. 34 (2), 21–38. Available online at: https://digital.car.chula.ac.th/aer/vol34/iss2/2/.

[B60] Qiu Y.-W. Qiu H.-L. Zhang G. Li J. (2019). Bioaccumulation and cycling of organochlorine pesticides (OCPs) and polychlorinated biphenyls (PCBs) in three mangrove reserves of south China. Chemosphere 217, 195–203. 10.1016/j.chemosphere.2018.10.188 30415117

[B61] Rodrigues A. S. Batista J. G. S. Rodrigues M. Á. V. Thipe V. C. Minarini L. A. R. Lopes P. S. (2024). Advances in silver nanoparticles: a comprehensive review on their potential as antimicrobial agents and their mechanisms of action elucidated by proteomics. Front. Microbiol. 15, 1440065. 10.3389/fmicb.2024.1440065 39149204 PMC11325591

[B62] Sharma V. K. Filip J. Zboril R. Varma R. S. (2015). Natural inorganic nanoparticles—Formation, fate, and toxicity in the environment. Chem. Soc. Rev. 44, 8410–8423. 10.1039/C5CS00236B 26435358

[B63] Sharma V. K. Sayes C. M. Guo B. Pillai S. Parsons J. G. Wang C. (2019). Interactions between silver nanoparticles and other metal nanoparticles under environmentally relevant conditions: a review. Sci. Total Environ. 653, 1042–1051. 10.1016/j.scitotenv.2018.10.411 30759545

[B64] Silva-Benavides A. M. (2009). “Mangroves,” in Marine biodiversity of Costa Rica, central America. Editors Wehrtmann I. S. Cortés J. (Springer), 123–130. 10.1007/978-1-4020-8278-8_7

[B65] Smita S. Gupta S. K. Bartonova A. Dusinska M. Gutleb A. C. Rahman Q. (2012). Nanoparticles in the environment: assessment using the causal diagram approach. Environ. Health 11 (Suppl. 1), S13. 10.1186/1476-069X-11-S1-S13 22759495 PMC3388445

[B66] Soni M. Pitchiah S. Suresh V. Ramasamy P. (2024). Fabrication and partial characterization of silver nanoparticles from mangrove (*Avicennia marina*) leaves and their antibacterial efficacy against oral bacteria. Cureus 16 (1), e52131. 10.7759/cureus.52131 38344562 PMC10858812

[B88] Souza-Júnior V. S. Vidal-Torrado P. Garcia-Gonzaléz M. T. Otero X. L. Macías F. (2008). Soil mineralogy of mangrove forests from the state of São Paulo, Southeastern Brazil. Soil Sci. Soc. Am. J. 72 (3), 848–857. 10.2136/sssaj2007.0197

[B67] Steingard C. H. Pinochet-Barros A. Wendel B. M. Helmann J. D. (2023). Iron homeostasis in *Bacillus subtilis* relies on three differentially expressed efflux systems. Microbiology 169 (1), 001289. 10.1099/mic.0.001289 36748638 PMC9993123

[B68] Suchomel P. Kvitek L. Panacek A. Prucek R. Hrbac J. Vecerova R. (2015). Comparative study of antimicrobial activity of AgBr and Ag nanoparticles (NPs). PLOS ONE 10 (3), e0119202. 10.1371/journal.pone.0119202 25781988 PMC4363559

[B69] Sulpis O. Agrawal P. Wolthers M. Munhoven G. Walker M. Middelburg J. J. (2022). Aragonite dissolution protects calcite at the seafloor. Nat. Commun. 13 (1), 1104. 10.1038/s41467-022-28711-z 35232971 PMC8888755

[B70] Taherkhani N. Piri H. Hekmat A. Haghbeen K. (2023). Humic and fulvic acids induced thermodynamic and structural instability of tyrosinase with antiproliferative effect on A375 melanoma cancer cell line. J. Inflamm. Dis. 26 (4), 183–192. 10.32598/JID.26.4.3

[B71] Tang D. Luo S. Deng S. Huang R. Chen B. Deng Z. (2022). Heavy metal pollution status and deposition history of mangrove sediments in zhanjiang Bay, China. Front. Mar. Sci. 9, 989584. 10.3389/fmars.2022.989584

[B72] Tang W. W. Zeng G. M. Gong J. L. Liang J. Xu P. Zhang C. (2014). Impact of humic/fulvic acid on the removal of heavy metals from aqueous solutions using nanomaterials: a review. Sci. Total Environ. 468–469, 1014–1027. 10.1016/j.scitotenv.2013.09.044 24095965

[B73] Tatzber M. Stemmer M. Spiegel H. Katzlberger C. Haberhauer G. Gerzabek M. H. (2008). Impact of different tillage practices on molecular characteristics of humic acids in a long-term field experiment—An application of three different spectroscopic methods. Sci. Total Environ. 406 (1–2), 256–268. 10.1016/j.scitotenv.2008.07.048 18789814

[B74] Vaish S. Pathak B. (2023). Mangrove synthesized bio-nanomaterial and its applications: a review. Environ. Nanotechnol. Monit. and Manag. 20, 100866. 10.1016/j.enmm.2023.100866

[B75] Vindedahl A. M. Strehlau J. H. Arnold W. A. Penn R. L. (2016). Organic matter and iron oxide nanoparticles: aggregation, interactions, and reactivity. Environ. Sci. Nano 3 (3), 494–505. 10.1039/C5EN00215J

[B76] Wang Y. Li D. Lu Z. Ma L. (2024). Decomposition and variation in carbon and nitrogen of leaf litter mixtures in a subtropical mangrove forest. Forests 15 (4), 672. 10.3390/f15040672

[B89] Wang Y. Zhang X. Bai Y. Li W. Li X. Xing X. (2020). Anticancer and antibacterial activities of silver nanoparticles (AgNPs) synthesized from cucumis melo L. J. Nanosci. Nanotechnol. 20 (7), 4143–4151. 10.1166/jnn.2020.17524 31968432

[B78] Warton B. Matthiessen J. N. (2005). The crucial role of calcium interacting with soil pH in enhanced biodegradation of metam-sodium. Pest Manag. Sci. 61 (9), 856–862. 10.1002/ps.1095 16010663

[B79] Wickham H. (2016). ggplot2: elegant graphics for data analysis. Springer. Available online at: https://ggplot2.tidyverse.org.

[B80] Willian N. Syukri Z. Labanni A. Arief S. (2020). Bio-friendly synthesis of silver nanoparticles using mangrove Rhizophora stylosa leaf aqueous extract and its antibacterial and antioxidant activity. Rasayan J. Chem. 13 (03), 1478–1485. 10.31788/rjc.2020.1335760

[B81] Wood S. N. (2011). Fast stable restricted maximum likelihood and marginal likelihood estimation of semiparametric generalized linear models. J. R. Stat. Soc. Series B. Stat. Methodol. 73 (1), 3–36 . 10.1111/j.1467-9868.2010.00749.x

[B82] Yin Y. Liu J. Jiang G. (2012). Sunlight-induced reduction of ionic Ag and Au to metallic nanoparticles by dissolved organic matter. ACS Nano 6 (9), 7910–7919. 10.1021/nn302293r 22816495

[B83] Zhang Z. Pei N. Sun Y. Li J. Li X. Yu S. (2019). Halogenated organic pollutants in sediments and organisms from mangrove wetlands of the jiulong river Estuary, south China. Environ. Res. 171, 145–152. 10.1016/j.envres.2019.01.028 30665116

