# Supplementary material for: Mangrove soil as a natural catalyst for green synthesis of silver nanoparticles
Source: Front Chem. 2025 Sep 5;13:1589836. doi: 10.3389/fchem.2025.1589836 (PMC12447646; doi:10.3389/fchem.2025.1589836)
Supplement: Supplementary file 1 [file DataSheet1.pdf]

frontiers

Supplementary Material

Mangrove Soil as a Natural Catalyst for Green Synthesis of Silver Nanoparticles.

Andrea Chacón-Calderón[1], Juan Miguel Zuñiga-Umaña[1,2], Claudia Villareal[1], José Roberto Vega-Baudrit[1,3], Reinaldo Pereira-Reyes[1], Yendry Corrales-Ureña[1]

1. National Laboratory of Nanotechnology LANOTEC - National Center of High Technology CeNAT, 1174-1200 Calle, San José, 10109, Costa Rica
2. Academic master's in bioinformatics and systems biology, Graduate Program in Biomedical Sciences, University of Costa Rica.
3. Escuela de Química, Universidad Nacional, Heredia, Costa Rica

- *** Correspondence:**
  Corresponding Author
  yendry386@hotmail.com; ycorrales@cenat.ac.cr

Methods:

Thermogravimetric Analysis (TGA)

The thermogravimetric analysis was performed using a TA Instruments Q500 analyzer. The samples were analyzed with a temperature ramp from 25 ºC to 1000 ºC at a heating rate of 10 ºC/min, using between 5 and 7 mg of each sample. The obtained thermograms were analyzed using the Universal Analysis software from TA Instruments and Origin software from OriginLab.

Results

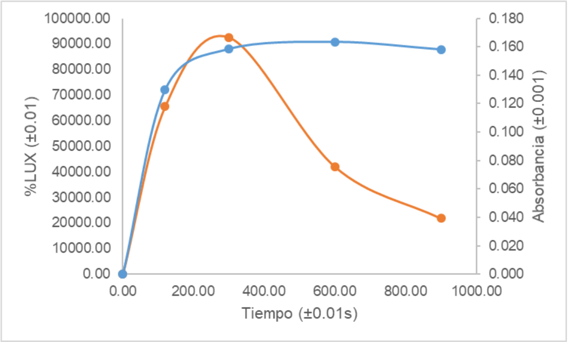

Cahuita 10 – 20 cm

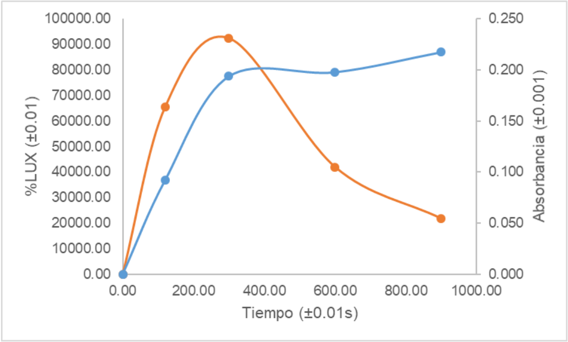

Cahuita 0 – 10 cm

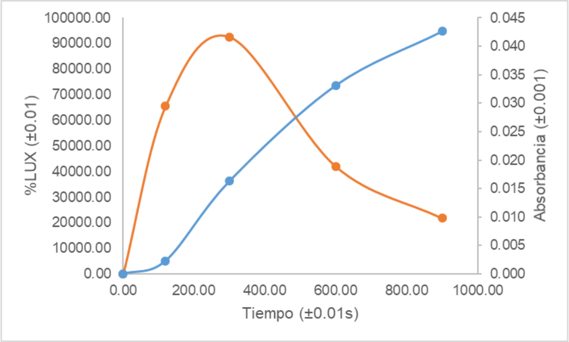

Punta Morales 10 – 20 cm

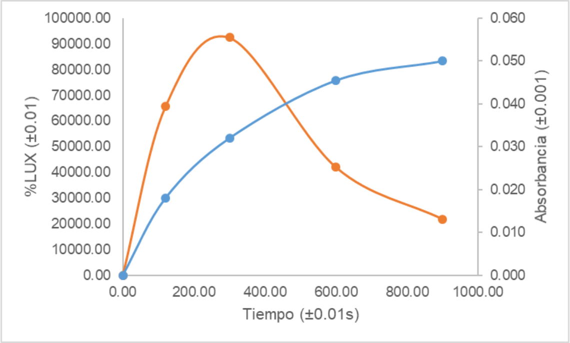

Punta Morales 0 – 10 cm

Supplementary Figure 1. Variation in absorbance and solar light intensity over time for each of the mangrove soil extracts during nanoparticle synthesis.

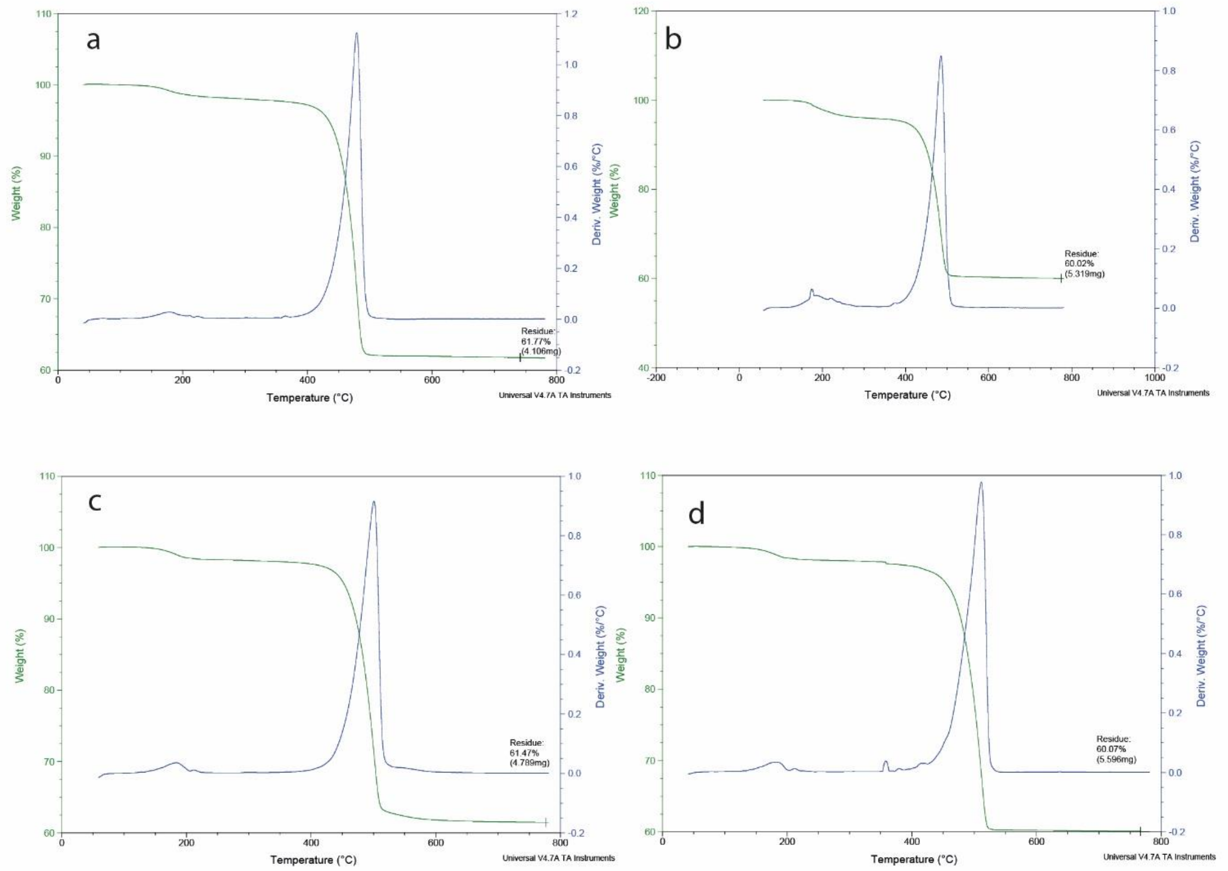

Supplementary Figure 2. Variation of the weight loss with respect to temperature (left Y axes) and the derivate of the weight with temperature (right Y axes) with temperature for the NPs synthesized using extracts: a) CI, b) CS, c) PMI, d) PMS.

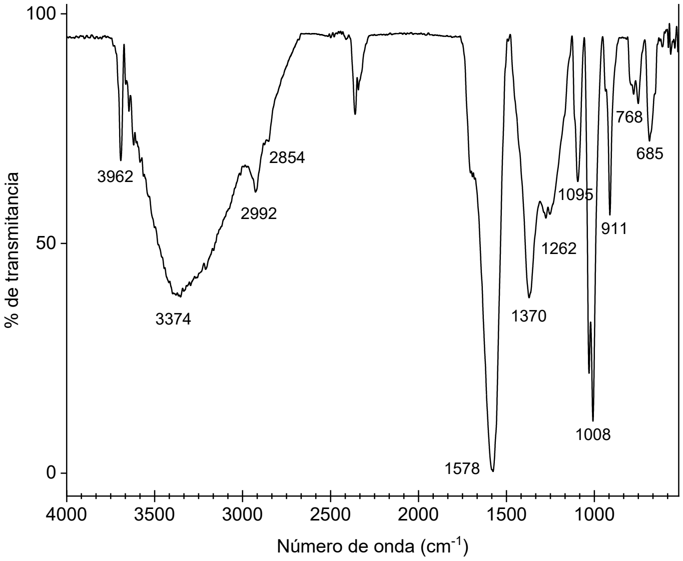

Supplementary Figure 3. ATR-FTIR spectra of humic acid reagent.

Supplementary Figure 4 shows the spectra from humic acid standard (Sigma Aldrich, USA). There are bands associated with carboxyl groups exhibit moderate to high intensity, such as those observed at 1370 cm^{-1} and 1578 cm^{-1}. Several similarities with the mangrove extract are identified, including the bands corresponding to O-H groups, amines, or thiols above 3370 cm^{-1}, the presence of aliphatic chains evidenced by the bands in the 2900-2800 cm^{-1} region, and the signals between 1100 and 1000 cm^{-1}, attributable to C-O bonds from polysaccharides. Nevertheless, one of the most significant aspects to highlight is that the bands in the spectrum of the solid standard are considerably more defined compared to those of the lyophilized extracts, which display broad bands with shoulders and strong overlap.

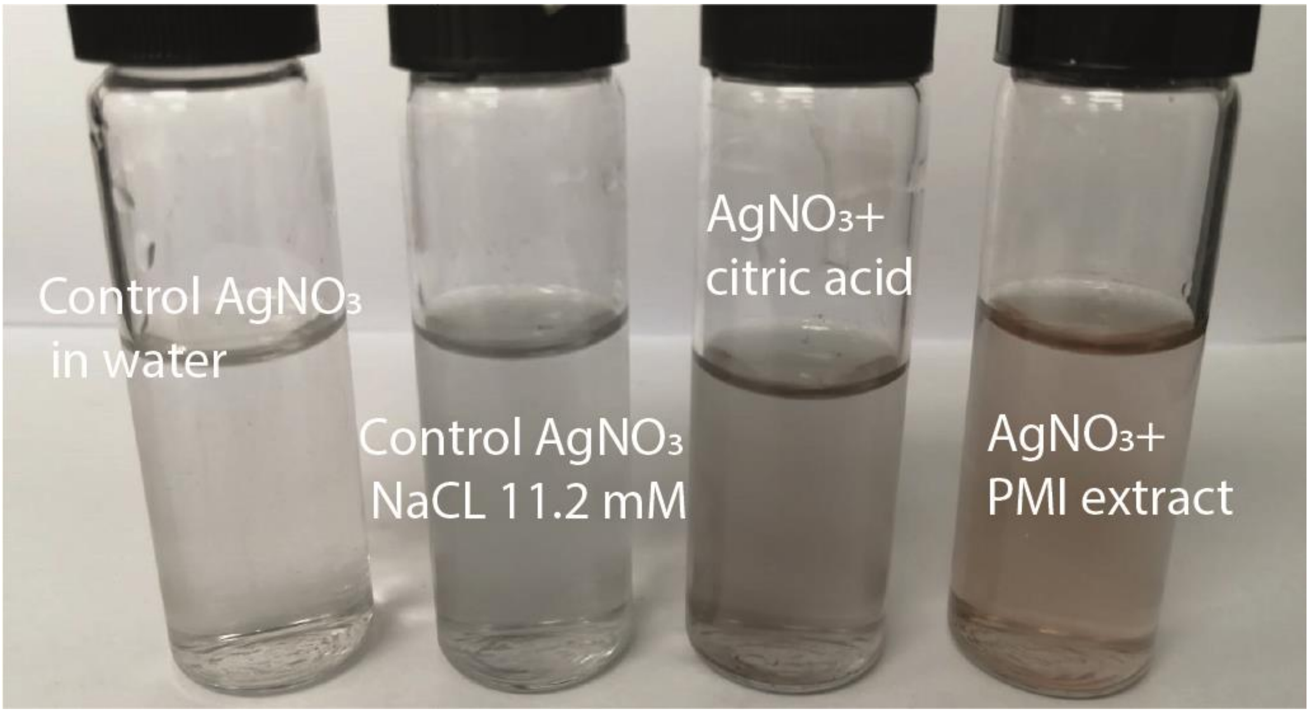

Supplementary Figure 4. Photographs of the control solutions used for one representative mangrove extract PMS, following nanoparticle exposure to sunlight for 15 minutes

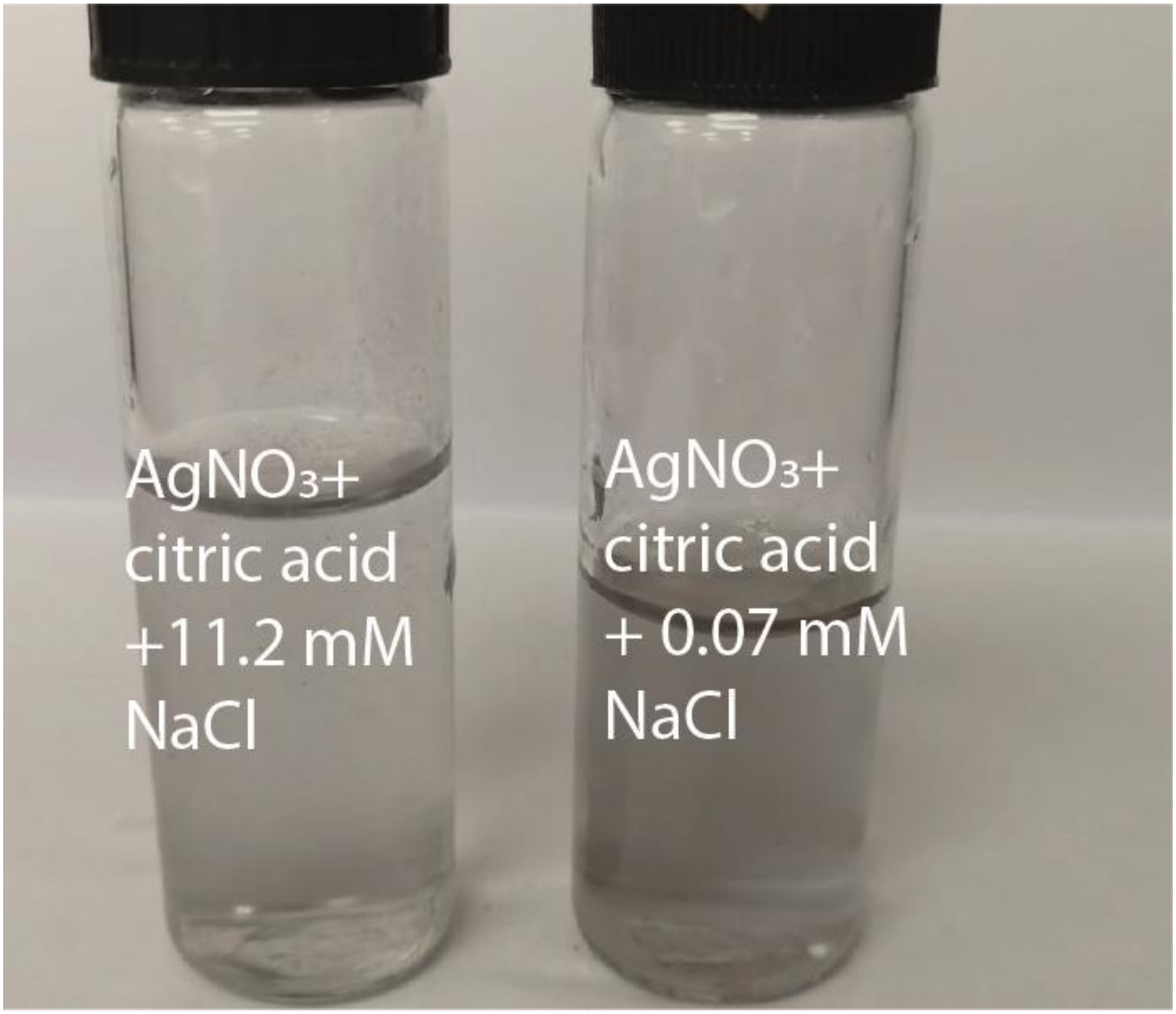

Supplementary Figure 5. Photographs of the of citric acid and $AgNO_3$ after exposure to sunlight for 15 min and different concentrations of NaCl.

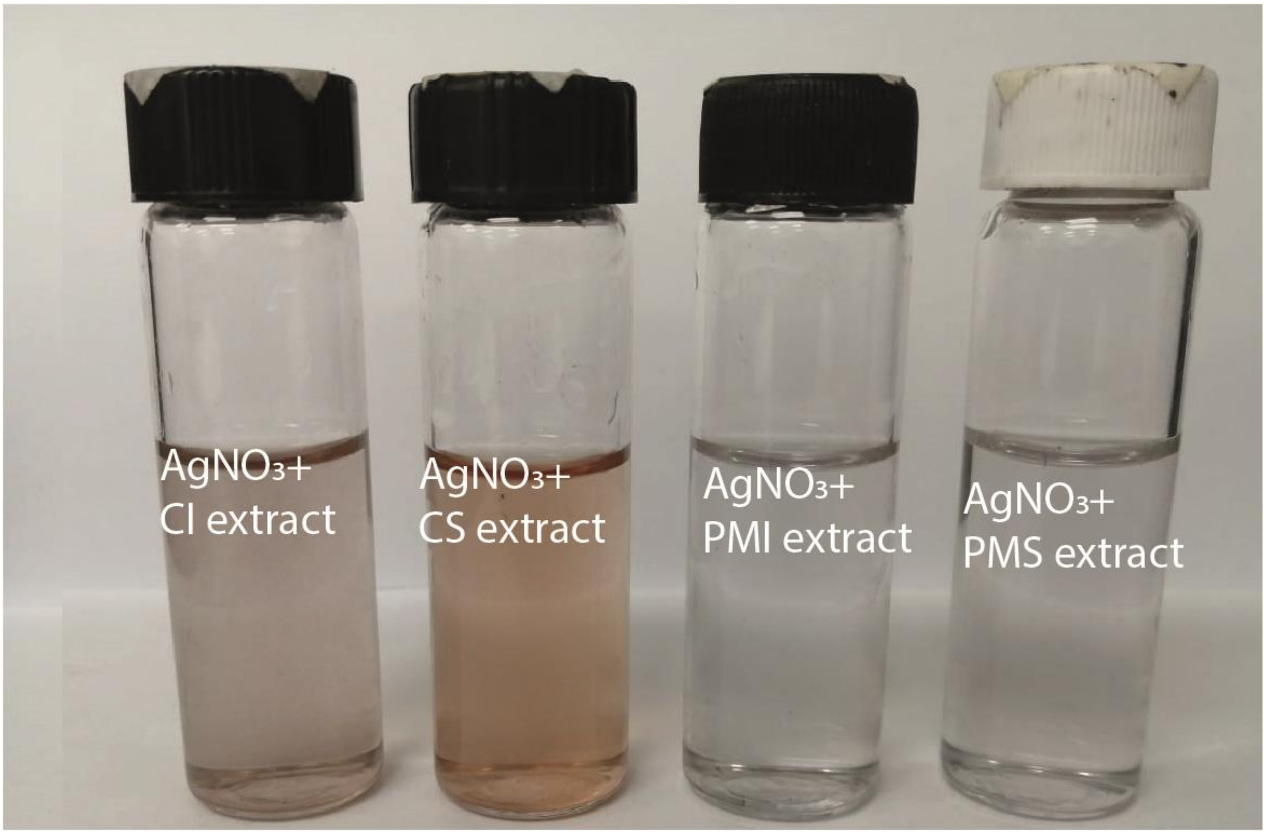

Supplementary Figure 6. Ag-NPs formation using mangrove extracts and sun irradiation for 15 min**.**

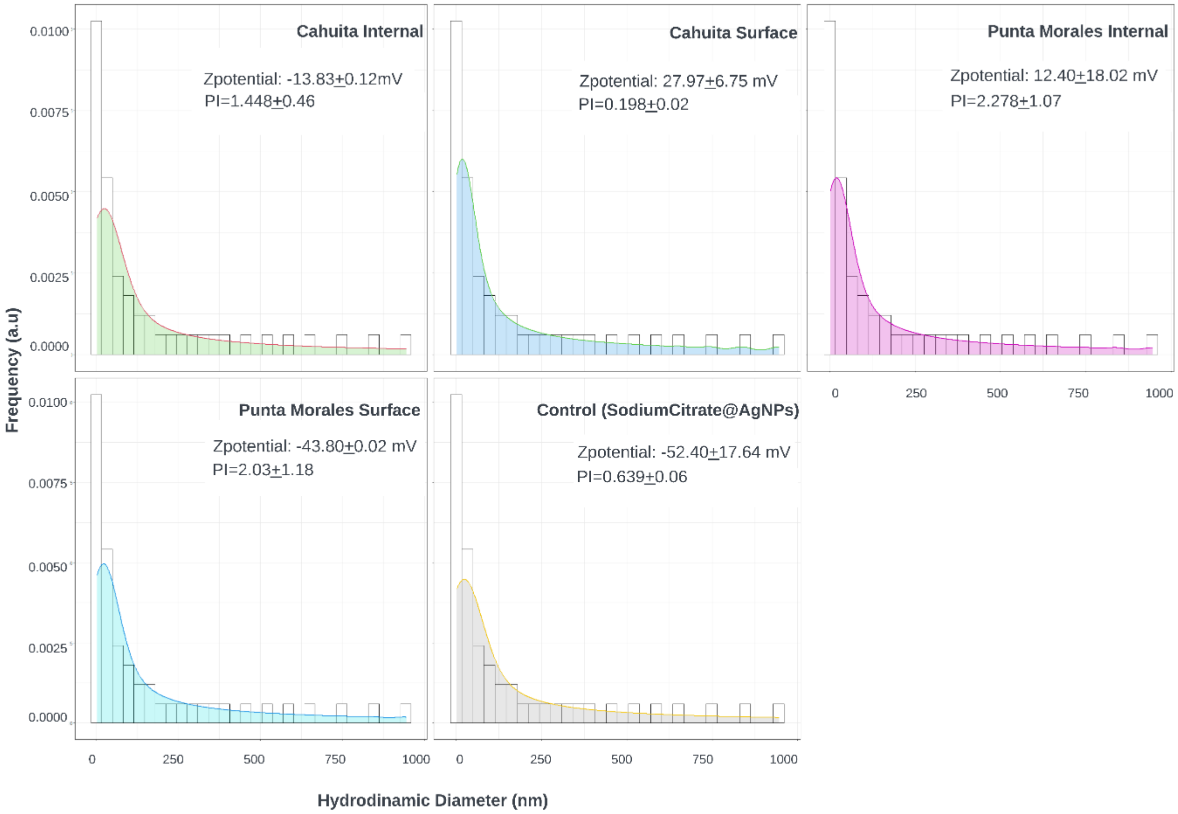

Supplementary Figure 7. Dynamic light scattering (DLS) results of the nanoparticles size distribution, zeta potential and polydispersity of the Ag NPs synthetized using the mangrove extract.

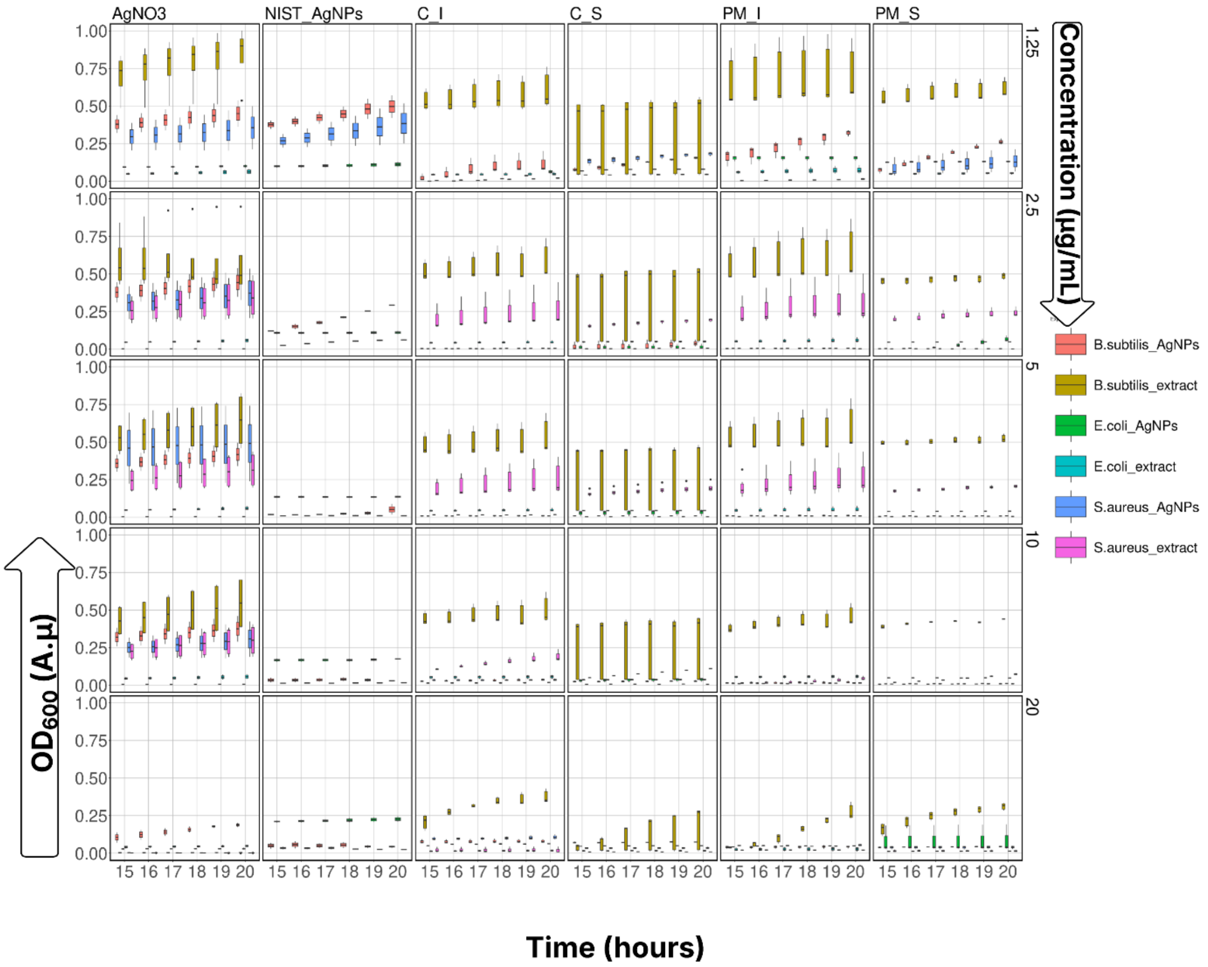

Supplementary Figure 8. Time-dependent variation in OD600 measurements between 15 and 20 hours for bacterial species subjected to treatment with extract or nanoparticles.

	Mangrove AgNPs												
	Dunn´s Post Hoc	**AgNO3**	**AgNPs**	**c+**	**C_I**	**C_S**	**PM_I**	**PM_S**	*Scheirer Ray Hare*	*Df*	*SumSq*	*H*	*P-value*
E.coli	**AgNO3**	NA							Concentration	4	6482667	298,526	**0**
	**AgNPs**	**0**	NA						Treatment	5	2291293	105,514	**0**
	**c+**	**0**	0,854	NA					Concent:Treatm	12	1024287	47,168	**4,36E-06**
	**C_I**	0,513	**0**	**0**	NA				Residual	489	2077450		
	**C_S**	**0**	**0**	**0**	**0**	NA							
	**PM_I**	**0,002**	**0**	**0**	**0,008**	0,261	NA						
	**PM_S**	**0**	**0**	**0**	**0**	0,883	0,343	NA					
B.subtilis	**AgNO3**	NA							Concentration	4	3533061	170,657	**0**
	**AgNPs**	**0**	NA						Treatment	5	3298183	159,309	**0**
	**c+**	0,161	**0**	NA					Concent:Treatm	20	1681560	81,224	**2,43E-08**
	**C_I**	**0**	**0**	**0**	NA				Residual	467	716940		0,000000
	**C_S**	**0**	**0,002**	**0**	0,166	NA							
	**PM_I**	**0**	**0**	**0**	0,867	0,203	NA						
	**PM_S**	**0**	**0**	**0**	0,164	**0,004**	0,124	NA					
S.aureus	**AgNO3**	NA							Concentration	4	1927726	93,217	**0**
	**AgNPs**	**0,018**	NA						Treatment	5	1663439	80,437	**6,66E-16**
	**c+**	**0,004**	**0**	NA					Concent:Treatm	20	5185463	250,747	**0,00E+00**
	**C_I**	**0**	**0**	**0**	NA				Residual	488			
	**C_S**	**0**	**0**	**0**	0,072	NA							
	**PM_I**	**0**	**0**	**0**	**0,008**	0,399	NA						
	**PM_S**	**0**	**0**	**0**	0,113	0,818	0,296	NA					

	Mangrove Extract											
	Dunn´s Post Hoc	**AgNO3**	**c+**	**C_I**	**C_S**	**PM_I**	**PM_S**	*Scheirer Ray Hare*	*Df*	*SumSq*	*H*	*P-value*
E.coli	**AgNO3**	NA						Concentration	5	3712413	167,126	**0**
	**AgNPs**							Treatment	5	3510500	158,037	**0**
	**c+**	**0**	NA					Concent:Treatm	16	1286493	57,916	**1,17E-06**
	**C_I**		**0**	NA				Residual	489	354066		
	**C_S**	**0**	**0**	0,062	NA							
	**PM_I**	0,784	**0**	**0**	**0**	NA						
	**PM_S**	**0**	**0**	0,06	0,972	**0**	NA					
B.subtilis	**AgNO3**	NA						Concentration	4	49115631	516,25	**0**
	**AgNPs**							Treatment	4	91841417	96,53	**0**
	**c+**	**0**	NA					Concent:Treatm	16	31740121	75,07	**1,27E-09**
	**C_I**		**0**	NA				Residual	1042			
	**C_S**	**0**	**0**	0,062	NA							
	**PM_I**	0,989	**0**	0,779	**0**	NA						
	**PM_S**	0,170	**0**	0,329	0,972	0,194	NA					
S.aureus	**AgNO3**							Concentration	4	6482667	298,526	**0**
	**AgNPs**							Treatment	5	2291293	105,514	**0**
	**c+**	**0**						Concent:Treatm	12	1024287	47,168	**1,36E-06**
	**C_I**	0,078	NA					Residual	488	2077450		
	**C_S**	**0,006**	**0**	**0**								
	**PM_I**	0,128	**0**	0,809	NA							
	**PM_S**	0,239	**0**	**0,006**	0,137	**0,011**	NA					

Supplementary Figure 9. Comparison of OD_{600} values between 15 and 20 hours of incubation for the three bacterial species analyzed. Contrast cell values represent Benjamini–Hochberg adjusted p-values for treatment comparisons ($AgNO_3$: silver nitrate control; AgNPs: NIST silver nanoparticle control; c^+: positive control; C_I: Cahuita Intern; C_S: Cahuita Surface; PM_I: Punta Morales Intern; PM_S: Punta Morales Surface). Statistically significant values are shown in bold. For simplicity, p-values less than 1e−04 are rounded to 0. Test parameter values are shown in the upper corner of each panel.

.

Mangrove extract

Fit: OD_{600} ~ *Treatment × Bacteria + s(Time, k = 4, by = Concentration)*

```
(Intercept) == 0                 1.551e-01  4.601e-03  33.706  < 0.001 ***
Treatmc_+ == 0                   1.287e-01  1.292e-02   9.959  < 0.001 ***
TreatmCahuita_Int == 0          -1.087e-02  7.095e-03  -1.533  0.94368
TreatmCahuita_Sup == 0          -2.899e-02  7.095e-03  -4.086  0.00111 **
TreatmPuntaMorales_Int == 0     -1.157e-02  7.095e-03  -1.630  0.90389
TreatmPuntaMorales_Sup == 0     -3.175e-02  6.965e-03  -4.558  < 0.001 ***
s(Time):Concent0.1 == 0          1.981e-02  2.256e-02   0.878  0.99996
s(Time):Concent0.2 == 0          3.737e-02  3.637e-02   1.028  0.99957
s(Time):Concent0.3 == 0          1.524e-01  2.318e-02   6.575  < 0.001 ***
s(Time):Concent0.03125.1 == 0 -2.504e-12  3.801e-06   0.000  1.00000
s(Time):Concent0.03125.2 == 0  7.558e-13  8.222e-06   0.000  1.00000
s(Time):Concent0.03125.3 == 0 -3.649e-12  3.895e-06   0.000  1.00000
s(Time):Concent0.0625.1 == 0   -1.530e-02  2.264e-02  -0.676  1.00000
s(Time):Concent0.0625.2 == 0    1.163e-01  2.402e-02   4.841  < 0.001 ***
s(Time):Concent0.0625.3 == 0    1.234e-01  2.160e-02   5.713  < 0.001 ***
s(Time):Concent0.125.1 == 0    -1.949e-04  1.752e-02  -0.011  1.00000
s(Time):Concent0.125.2 == 0     8.648e-02  1.945e-02   4.446  < 0.001 ***
s(Time):Concent0.125.3 == 0     9.219e-02  1.672e-02   5.513  < 0.001 ***
s(Time):Concent0.25.1 == 0      3.946e-04  1.389e-02   0.028  1.00000
s(Time):Concent0.25.2 == 0      5.689e-02  1.642e-02   3.463  0.01271 *
s(Time):Concent0.25.3 == 0      8.774e-02  1.338e-02   6.557  < 0.001 ***
s(Time):Concent0.5.1 == 0       5.297e-02  1.266e-02   4.183  < 0.001 ***
s(Time):Concent0.5.2 == 0      -1.777e-03  1.919e-02  -0.093  1.00000
s(Time):Concent0.5.3 == 0       2.921e-02  1.155e-02   2.530  0.22907
s(Time):Concent1.1 == 0         6.195e-03  1.230e-02   0.504  1.00000
s(Time):Concent1.2 == 0        -3.904e-02  1.774e-02  -2.201  0.46472
s(Time):Concent1.3 == 0         2.157e-02  1.196e-02   1.803  0.79985
```

AgNPs

Fit: OD_{600} ~ *Treatment × Concentration + s(Time, k = 4, by = Bacteria)*

```
                                      Estimate Std. Error t value Pr(>|t|)
(Intercept)                           0.374197   0.017429  21.470  < 2e-16 ***
TreatmAgNPs_                          0.014726   0.024648   0.597 0.550305
TreatmCahuita_Int                    -0.322628   0.022501 -14.339  < 2e-16 ***
TreatmCahuita_Sup                    -0.216140   0.022501  -9.606  < 2e-16 ***
TreatmPuntaMorales_Int               -0.195367   0.022501  -8.683  < 2e-16 ***
TreatmPuntaMorales_Sup               -0.188306   0.022501  -8.369  < 2e-16 ***
Concent0.125                         -0.036832   0.024648  -1.494 0.135318
Concent0.25                           0.015888   0.024648   0.645 0.519306
Concent0.5                           -0.091465   0.024648  -3.711 0.000215 ***
Concent1                             -0.292401   0.024648 -11.863  < 2e-16 ***
TreatmAgNPs_:Concent0.125            -0.191301   0.034858  -5.488 4.81e-08 ***
TreatmCahuita_Int:Concent0.125       -0.008314   0.031821  -0.261 0.793918
TreatmCahuita_Sup:Concent0.125       -0.100041   0.031821  -3.144 0.001702 **
TreatmPuntaMorales_Int:Concent0.125 -0.135256   0.031821  -4.251 2.27e-05 ***
TreatmPuntaMorales_Sup:Concent0.125 -0.133434   0.031821  -4.193 2.92e-05 ***
TreatmAgNPs_:Concent0.25             -0.327935   0.034858  -9.408  < 2e-16 ***
TreatmCahuita_Int:Concent0.25        -0.052788   0.031821  -1.659 0.097354 .
TreatmCahuita_Sup:Concent0.25        -0.154355   0.031821  -4.851 1.37e-06 ***
TreatmPuntaMorales_Int:Concent0.25   -0.183055   0.031821  -5.753 1.08e-08 ***
TreatmPuntaMorales_Sup:Concent0.25   -0.192827   0.031821  -6.060 1.75e-09 ***
TreatmAgNPs_:Concent0.5              -0.198818   0.034858  -5.704 1.43e-08 ***
TreatmCahuita_Int:Concent0.5          0.078026   0.031821   2.452 0.014325 *
TreatmCahuita_Sup:Concent0.5         -0.035750   0.031821  -1.123 0.261422
TreatmPuntaMorales_Int:Concent0.5    -0.067024   0.031821  -2.106 0.035354 *
TreatmPuntaMorales_Sup:Concent0.5    -0.081215   0.031821  -2.552 0.010807 *
TreatmAgNPs_:Concent1                 0.038028   0.034858   1.091 0.275484
TreatmCahuita_Int:Concent1            0.348572   0.031821  10.954  < 2e-16 ***
TreatmCahuita_Sup:Concent1            0.205389   0.031821   6.455 1.49e-10 ***
TreatmPuntaMorales_Int:Concent1       0.176393   0.031821   5.543 3.54e-08 ***
TreatmPuntaMorales_Sup:Concent1       0.181860   0.031821   5.715 1.34e-08 ***
---
Signif. codes:  0 '***' 0.001 '**' 0.01 '*' 0.05 '.' 0.1 ' ' 1
```

Supplementary Figure 10. Output for GLM on mangrove extract and AgNPs.
